# Communications – I International Congress of the Scientific Society of Psychoneuroimmunology (SOCIPNI), Granada, November 13–14, 2025

**DOI:** 10.31083/RN47949

**Published:** 2025-12-25

## 1. Symposiums

**S1. Perinatal Mental Health and Its Consequences for Mothers and Offspring**

**Lecture 1. Perinatal Anxiety And Stress, Psychoneuroimmunological Factors, Body Mass Index (BMI) and Risk of Psychopathological Symptoms in a Sample of Spanish Pregnant Women**

Gloria Sánchez-Torices^1^, Jesús Joaquín Hijona^2^, María Isabel Peralta-Ramirez^3^, Teresa Sánchez-Gutiérrez^4^

^1^Faculty of Health Science, Universidad Internacional de La Rioja (UNIR), 26006 Logroño, Spain

^2^Obstetric and Gynecological Department, Complejo Hospitalario de Jaén, 23007 Jaén, Spain

^3^Psychology School, University of Granada, 18071 Granada, Spain

^4^Department of Psychology, University of Cordoba, 14071 Córdoba, Spain

**Introduction**: Perceived perinatal anxiety (PPA) and stress have shown to affect maternal health. However, the psychoneuroimmunological risk factors associated with possible psychopathological symptoms in pregnant women are not conclusive yet. The objtevies were; 1) to analyze if PPA, stress levels and psychoneuroimmunological factors [hair cortisol levels (HCL) and interleukin-6 levels (IL-6)] increase the likelihood for psychopathological symptoms in pregnant women; 2) to compare the levels of PPA, stress, psychopathological symptomatology and psychoneuroimmunological factors in pregnant women regarding their IMC levels. **Methodology**: A total of N = 70 women in their third trimester of pregnancy (29-41 weeks) were recruited at the Hospital of Jaen (Spain). We collected sociodemographical and clinical variables through clinical records; levels of perinatal anxiety and stress with the PASS and NuPDQ, and biological samples (HCL and IL-6). U-Mann Whitney, linear and binary regressions were used. We included age, number of miscarriages and first time pregnancy as covariates. **Results**: Higher PPA and stress were associated with higher scores on psychopathological symptoms, after controlling for the covariables: total score (PASS: B = 1.0, t = 7.7, *p* < 0.001; NuPDQ: B = 1.2, t = 3.1, *p* = 0.003; R^2^ = 0.7), hostility (PASS: B = 0.07, t = 3.5, *p* < 0.001; R^2^ = 0.3), somatization (PASS: B = 0.07; t = 2.3, *p* = 0.022; NuPDQ: B = 0.2; t = 2.3, *p* = 0.023, R^2^ = 0.4); depression (PASS: B = 0.2; t = 5.5, *p* < 0.001; NuPDQ: B = 0.2; t = 2.6, *p* = 0.017, R^2^ = 0.5); obsessive-compulsive (PASS: B = 0.2; t = 5.7, *p* < 0.001; R^2^ = 0.5); anxiety (PASS: B = 0.1; t = 5.7, *p* < 0.001; NuPDQ: B = 0.2; t = 32.3, *p* = 0.001, R^2^ = 0.5); sensitivity (PASS: B = 0.2; t = 6.1, *p* = 0.022; R^2^ = 0.5); phobic anxiety (NuPDQ: B = 0.1; t = 2.3, *p* = 0.025, R^2^ = 0.2), paranoid ideation (PASS: B = 0.1; t = 5.2, *p* < 0.001; R^2^ = 0.4) and psychoticism (PASS: B = 0.06; t = 5.4, *p* < 0.001; NuPDQ: B = 0.2; t = 2.6, *p* = 0.017, R^2^ = 0.4). Moreover, HCL was significantly associated with depressive symptoms (B = –2.2; t = –3.4, *p* = 0.001; R^2^ = 0.4) and IL-6 was significantly associated with paranoid ideation (B = –1.2; t = –2.1, *p* = 0.043; R^2^ = 0.4). Comparisons on perinatal anxiety and stress, psychopathological symptoms and psychoneuroimmunological factors by levels of IMC showed no significant differences, neither in the total score nor in the psychopathological subscales. However, we observed that the group with higher IMC presented significantly more levels of IL-6 (U = 349.0; *p* = 0.021) and count of B lymphocytes (U = 351.5; *p* = 0.048). **Conclusions**: Women with PPA or stress during their third trimester of pregnancy are at a greater risk of developing psychopathological symptoms than those who do not report this condition.

**Lecture 2. Pregnancy and Emotions: How Maternal Depression Shapes Children’s Temperament and Behavior**

J De Echarri-Lorente^1,2^, M.A Baos-González^1,2^, M.I. Peralta Ramirez^1,2^

^1^Department of Personality, Assessment, and Psychological Treatments, Faculty of Psychology, University of Granada, 18071 Granada, Spain

^2^Mind, Brain and Behavior Research Center (CIMCYC), University of Granada, 18071 Granada, Spain

**Introduction**: Prenatal period has significant influences on fetal and child developmental variables. One such variable is temperament, defined as stable individual differences in behavior and emotional reactions that emerge in the early years of life. Previous research has shown that higher levels of maternal stress during pregnancy and other mental health problems predict children with temperaments characterized by high negative emotionality and low self-regulation. Also, children characterized as high temperamental emotionality are more vulnerable to develop psychopathology in highly vulnerable environments. The aim of this lecture is to examine the relationship between maternal depression during pregnancy, the presence of emotional temperament in children aged 2 to 5 years, and its association with psychopathological problems. **Methodology**: The sample consisted of 99 mother–child dyads at age 2, 68 at age 3, 42 at age 4, and 33 at age 5, all from the Gestastress-Childstress cohort. Maternal depression during pregnancy was assessed using the Symptom Checklist-90-R (SCL-90-R), child temperament with the EAS scale, and child psychopathology with the CBCL. **Results**: Findings indicated that maternal depression predicted a temperament characterized by high emotionality in children aged 2 to 5. In turn, this type of temperament showed a strong association with both internalizing and externalizing problems across all ages. **Conclusions**: These results highlight that maternal mental health problems during pregnancy influence children’s emotional regulation, representing a significant risk factor for the development of psychopathological problems in early childhood. Developing programs to protect from stress, depression and anxiety during pregnancy is crucial.

**Lecture 3. Sex-Specific Pathways Linking Maternal Mental Health and Early Psychopathology To Stress Reactivity**

M.A Baos-González^1,2^, J De Echarri-Lorente^1,2^, M.I. Peralta Ramirez^1,2^

^1^Department of Personality, Assessment, and Psychological Treatments, Faculty of Psychology, University of Granada, 18071 Granada, Spain

^2^Mind, Brain and Behavior Research Center (CIMCYC), University of Granada, 18071 Granada, Spain

**Introduction**: Atypical stress reactivity in early life has been linked to long-term socioemotional and health outcomes. This study examined whether maternal perinatal mental health and child psychopathology at age two predicted stress reactivity at ages four to five, also exploring sex differences. **Methodology**: Forty-two mother–infant dyads participated. During pregnancy, mothers completed psychological questionnaires and provided hair samples in each trimester. Mental health was assessed with the Pregnancy Distress Questionnaire (PDQ), Perceived Stress Scale (PSS), hair cortisol concentration (HCC, log-transformed), and anxiety, phobic anxiety, and depression subscales of the Symptom Checklist-90-R (SCL-90-R). Mean pregnancy values were calculated. At age two, 31 mothers completed the CBCL. At ages four to five, stress reactivity was assessed with the Stress Reactivity Task for Preschoolers, calculating area-under-the-curve indices (AUCg, AUCi) for salivary cortisol and alpha-amylase. Pearson correlations and linear regressions tested associations, considering sex differences. **Results**: Maternal perinatal mental health variables were not significantly correlated with stress reactivity overall at age four, but distinct associations emerged when boys and girls were analysed separately. Child psychopathology at age two showed significant links: Somatic Complaints were negatively correlated with cortisol AUCg (r = –0.440, *p* = 0.015), Oppositional Defiant Problems were positively correlated with cortisol AUCi (r = 0.367, *p* = 0.046), and Aggressive Behaviour and ADHD Problems were positively correlated with alpha-amylase AUCg (r = 0.417, *p* = 0.020; r = 0.389, *p* = 0.030). Regression models confirmed these effects (Somatic Complaints: R^2^ = 0.194, β = –0.440, *p* = 0.015; Oppositional Defiant Problems: R^2^ = 0.135, β = 0.367, *p* = 0.046; Aggressive Behaviour–ADHD: R^2^ = 0.203, *p* = 0.041). Several of these associations differed between boys and girls. **Conclusions**: Preschoolers’ stress reactivity was related to maternal perinatal stress only when analyzed by sex, while early psychopathology at age two predicted stress reactivity at age four to five with distinct patterns for boys and girls.

**S2. Nutrition and the Mind-Gut Connection: Emerging Insights in Psychoneuroimmunology**

**Lecture 1. Fasting and the Gut-Brain Axis: A Holistic Pathway to Healthy Longevity**

Robin Mesnage^1,2^

^1^Buchinger Wilhelmi Clinic, 88662 Überlingen, Germany

^2^Department of Nutritional Sciences, School of Life Course Sciences, Faculty of Life Sciences and Medicine, King’s College London, SE1 9NH London, UK

**Introduction**: Fasting is increasingly applied as a dietary intervention, with protocols differing in duration and composition. Buchinger therapeutic fasting, a modified regimen providing ~250 kcal/day, has been practiced in clinical settings for nearly a century. Clinical studies at the Buchinger Wilhelmi Clinics have explored its physiological and psychological effects. **Methodology**: The standardized program involves 4–21 days of modified fasting (~250 kcal/day from vegetable broths, fruit juices, and honey), followed by controlled food reintroduction. Participants are medically supervised, with regular monitoring of vital signs, anthropometry, and laboratory markers. All studies received ethical approval, and participants provided informed consent. **Results**: Buchinger fasting induces coordinated adaptations along the microbiota–gut–brain axis. In the intestine, nutrient-dependent bacteria decline, while taxa metabolizing host-derived substrates expand, resembling metabolic reprogramming during hibernation. These shifts occur without barrier disruption and coincide with enhanced mucosal immunity, including increased secretory immunoglobulin A. Upon food reintroduction, microbial diversity and short-chain fatty acid production are restored, supporting homeostasis. Neuroimaging demonstrates preserved brain structure during prolonged fasting. At the molecular level, Buchinger fasting reduces oxidative stress, strengthens antioxidant defenses, and activates autophagy, promoting protein clearance, synaptic plasticity, and neurogenesis. These processes translate into improved emotional well-being, modulation of autonomic activity, and greater cognitive resilience. Psychologically, fasting introduces a psychosomatic dimension, as food withdrawal—a strong emotional stimulus—encourages introspection and can facilitate psychotherapeutic interventions.

**Conclusion**: Overall, Buchinger therapeutic fasting emerges as a safe, multifaceted intervention modulating the microbiota–gut–brain axis, with potential to support chronic disease prevention and healthy aging.

**Lecture 2. Diet, Microbiota, and Mood: Mapping Associations in a Multi-Omics Framework**

Stefanie Malan-Müller^1,2,3,4^, Leire Virto Ruiz^5,6,^, Iñaki Zorrilla^2,7^, Javier de Diego-Adeliño^2,8,9,10^, Marta Cano^2,8^, Maria Paz García-Portilla^2,11^, Ana González-Pinto^2,7^, Juan C. Leza^1,2,3,4^

^1^Department of Pharmacology and Toxicology, Faculty of Medicine, University Complutense Madrid (UCM), 28040 Madrid, Spain

^2^Biomedical Research Network Centre in Mental Health (CIBERSAM), Institute of Health Carlos III (ISCIII), 28029 Madrid, Spain

^3^Hospital 12 de Octubre Research Institute (Imas12), 28041 Madrid, Spain

^4^Instituto Universitario de Investigación Neuroquímica (IUIN-UCM), 28040 Madrid, Spain

^5^ETEP (Etiology and Therapy of Periodontal and Peri-implant Diseases) Research Group, University Complutense Madrid (UCM), 28040 Madrid, Spain

^6^Department of Dental Clinical Specialties, Faculty of Dentistry, University Complutense Madrid (UCM), 28040 Madrid, Spain

^7^BIOARABA, Department of Psychiatry, Hospital Universitario de Alava, UPV/EHU, 01009 Vitoria, Spain

^8^Sant Pau Mental Health Research Group, Institut de Recerca Sant Pau (IR Sant Pau), 08041 Barcelona, Spain

^9^Hospital de la Santa Creu i Sant Pau, 08025 Barcelona, Spain

^10^Autonomous University of Barcelona (UAB), 08193 Barcelona, Spain

^11^Department of Psychiatry, Universidad de Oviedo, Servicio de Psiquiatría, Oviedo, Instituto de Investigación Sanitaria del Principado de Asturias (ISPA), 33011 Oviedo, Asturias, Spain

**Introduction**: Diet and the gut microbiome are increasingly recognized as modulators of mental health through pathways involving inflammation, metabolic activity, and microbial metabolites. This study aimed to characterize gut microbiota, metabolite, and immune profiles associated with mental health outcomes and dietary patterns in a Spanish population cohort. **Methodology**: We examined fecal microbiomes of individuals reporting mental health symptoms (n = 218) and mentally healthy controls (n = 66) using 16S rRNA sequencing. Plasma metabolites (n = 86), including SCFAs, indoles, amino acids, choline oxidation, kynurenine pathway intermediates, and B-vitamer forms, were quantified using GC-MS/MS and LC-MS/MS. Inflammatory and endothelial markers (CD31, HSP60, IL-1B, IL-4, IL-6, IL-10, NECTIN2, TNFA) were measured using a multiplex Luminex assay. Mental health symptoms were assessed with validated questionnaires. Dietary intake was captured via an Indicator food list (qualitative dietary patterns) and the STEPS survey for fruit and vegetable consumption. **Results**: A diagnosis of depression was associated with higher relative abundance of *Coprococcus catus*, while trauma-related variables, including PTSD symptoms and childhood trauma scores, were linked to higher levels of *Desulfovibrio piger*. Overall quality of life was positively associated with *Gemmiger qucibialis*, *Bifidobacterium*, and *Ruminococcus callidus*, and inversely associated with *Anaerotruncus colihominis*. Immune profiling revealed elevated plasma IL-1B, IL-6, and LPS, alongside reduced CD31, in individuals reporting mental health symptoms. Direct associations between dietary intake and mental health outcomes were limited, although higher consumption of whole grains and nuts/seeds/legumes correlated with better quality of life. Notably, these plant-rich dietary patterns were also associated with enrichment of SCFA-producing taxa, favorable metabolite profiles (including indole-3-propionic acid and B-vitamin forms), and improved markers of one-carbon metabolism, suggesting potential modulation of inflammatory processes and gut–brain pathways. **Conclusions**: Our findings indicate that dietary patterns may influence mental health indirectly by shaping gut microbiota and metabolite profiles relevant to immune and inflammatory processes. These results support the potential of plant-rich diets in promoting gut-brain health, though causality cannot be inferred due to the cross-sectional design.

**Lecture 3. Ultra-Processed Foods, Mental and Brain Health: A Translational View**

Adam Alvarez-Monell^1^, Sílvia Fernández-Barrès^2^, Carles Biarnés^3^, Anna Motger-Albertí^4^, Verónica Palomera-Ávalos^1,5^, Gerard Blasco^3^, Josep Puig^3^, José Manuel Fernández-Real^4,6,7,8^, Montserrat Solanas^5,9^, Rosa M Escorihuela^1,5^, Oren Contreras-Rodríguez^1,5,10,11^

^1^Department of Psychiatry and Forensic Medicine, Universitat Autònoma de Barcelona (UAB), 08193 Barcelona, Spain

^2^Agència de Salut Pública de Barcelona, 08023 Barcelona, Spain

^3^Medical Imaging Research Group-IDI, Girona Biomedical Research Institute (IdIBGi), 17190 Girona, Spain

^4^Department of Nutrition, Eumetabolism, and Health, Girona Biomedical Research Institute (IdibGi), Josep Trueta University Hospital, 17190 Girona, Spain

^5^Neuroscience Institute, Universitat Autònoma de Barcelona (UAB), 08193 Barcelona, Spain

^6^Department of Medical Sciences, School of Medicine, University of Girona, 17003 Girona, Spain

^7^CIBER Fisiopatología de la Obesidad y Nutrición (CIBEROBN), 28029 Madrid, Spain

^8^Instituto de Salud Carlos III (ISCIII), 28029 Madrid, Spain

^9^Department of Cell Biology, physiology and Immunology, Universitat Autònoma de Barcelona (UAB), 08193 Barcelona, Spain

^10^CIBER de Salud Mental (CIBERSAM), 28029 Madrid, Spain

^11^Unitat Mixta de Neurociència Traslacional I3PT-Autonomous, Universitat Autònoma de Barcelona, 08193 Barcelona, Spain

**Introduction**: Ultra-processed foods and drinks (UPFs) result from intensive industrial processing and are characterized by low nutrient and high energy density. Their consumption has been associated with deteriorated mental health and increase inflammation. We aim to investigate associations between UPF intake, brain features, depressive symptoms and inflammatory markers. **Methodology**: 150 healthy adult subjects completed dietary assessments (food frequency questionnaire), depressive symptom screening (PHQ-9), laboratory tests, and brain MRI scans. Appropriate statistics explored relationships between UPF intake, depressive symptoms, brain features (gray matter brain volumes and perfusion) and inflammatory markers (e.g., total leukocytes, lymphocytes, monocytes), considering obesity status. In parallel, 49 Long-Evans rats were fed either standard chow (STD) or a novel UPF diet from prenatal or postweaning periods and underwent resting-state MRI at postnatal days 60-67. **Results**: In humans, higher UPF intake was positively associated with depressive symptoms and elevated inflammatory markers. Greater UPF consumption was linked to reduced volume but increased perfusion in the anterior cingulate cortex and amygdala, with some effects moderated by obesity. Total leukocytes and lymphocyte counts significantly mediated the relationship between UPF intake, depressive symptoms, and perfusion in the anterior cingulate cortex. In the animal model, rats exposed to the UPF diet showed increased functional connectivity in brain regions analogous to the anterior cingulate cortex. **Conclusion**: UPF intake is associated with structural and functional brain changes in regions relevant to mood regulation, both in humans and in animal models. Inflammatory markers play a key mediating role, highlighting potential biological pathways linking diet to mental health.

**S3. Psychoneuroimmunological Implications of Intimate Partner Violence Against Women**

**Lecture 1. Relationship Between Trauma and Immunological Functioning: Intimate Partner Violence Survivors**

Andrea Benítez Quintana^1^, Noelia Pérez Cámara^1^, Julia Hernández Cano^1^, Eva María Rodríguez Félix^1^, Carmen Fernández Fillol^1^, Miguel Pérez García^1^

^1^University of Granada, 18012 Granada, Spain

The association between psychological trauma and the immune system has been well documented. In acute threat situations, activation of the sympathetic nervous system (SNS) and the hypothalamic-pituitary-adrenal (HPA) axis facilitates the release of catecholamines and cortisol, thereby complementarily modulating immune responses. This interplay is adaptive in the short term. When stress becomes chronic, these systems gradually lose their regulatory efficacy, leading to receptor desensitization in the immune system and the emergence of chronic inflammation at central and peripheral levels. This altered physiological state increases the organism’s vulnerability to a wide range of disorders. Accordingly, post-traumatic stress disorder (PTSD) is strongly associated with immune alterations. In this sense, meta-analyses report increased IL-6, TNF-α, and IFN-γ and reduced IL-10, consistent with a pro-inflammatory immune profile. For example, in different trauma-exposed populations early-life adversity leaves long-lasting biological traces, predicting higher CRP, IL-6, and TNF-α in adulthood. In refugees and war survivors, several studies link accumulated trauma with systemic inflammation. On the other hand, Intimate Partner Violence Against Women (IPVAW) is a unique form of trauma, marked by repeated and intentional harm within an emotional bond. Survivors often develop not only classical PTSD but also complex PTSD, reflecting its chronic nature. Evidence indicates that Intimate Partner Violence (IPV) is associated with neural disruptions and immune alterations, including dysregulated cortisol, altered glucocorticoid sensitivity, impaired lymphocyte function, reduced salivary IgA, and elevated pro-inflammatory cytokines. These changes weaken antiviral defenses and increase vulnerability to disease. Overall, trauma leaves a lasting immunological imprint with clinical consequences.

**Lecture 2. Relationship Between Immunological Indexes and Clinical Conditions in Women Survivors of Intimate Partner Violence**

María Pérez González^1^, Julia Arnal de la Peña^1^, María Dolores Sánchez Rodríguez^1^, Luz Stella Algarra López^1^, Raquel González Pérez^1^, Carla Camacho González^1^, Juan Verdejo Román^1^

^1^University of Granada, 18012 Granada, Spain

**Introduction**: Systemic inflammation constitutes a broad and intricate physiological response triggered by the organism when exposed to harmful stressors such as traumas or chronic conditions and it has also been reported in women survivors of Intimate Partner Violence Against Women (IPVAW). **Methodology**: A total of 63 women participated in the study: 36 were survivors of IPVAW and 27 non-IPVAW victims. All participants attended a psychopathological assessment session where they completed the GAD-7 questionnaire to assess anxiety, Perceived Stress Scale (PSS) to assess perceived stress and the severity of the IPVAW questionnaire. Moreover, a blood collection session was also carried out to evaluate biological indicators such as the neutrophil-to-lymphocyte ratio (NLR), platelet-to-lymphocyte ratio (PLR), monocyte-to-lymphocyte ratio (MLR), the systemic immune-inflammation index (SII), the systemic inflammation response index (SIRI). The SII is a composite index that integrates platelets, neutrophils, and lymphocytes to provide a comprehensive measure of systemic inflammation, reflecting the balance between pro-inflammatory and anti-inflammatory immune components. Similar to SII, SIRI is an integrated inflammatory biomarker that combines monocytes, neutrophils, and lymphocytes. **Results**: Women survivors of IPVAW showed higher punctuations in anxiety (*p* < 0.001) and perceived stress (*p* < 0.001). No significant differences were found when examining immunological indexes in the IPVAW group compared to the control group. Furthermore, partial correlations controlling for age were conducted in the IPVAW group, where all immunological indexes (NLR, PLR, MLR, SIRI and SII) showed a negative relationship with anxiety (*p* < 0.01), perceived stress (*p* < 0.05), and the severity of violence experienced more than one year ago (*p* < 0.05). **Conclusions**: These results are contrary to the reviewed literature in other clinical populations, suggesting that future research should continue exploring immunological indexes and their relationship to clinical variables in women survivors of IPVAW.

**Lecture 3. Psychoneuroendocrinoimmunological Diseases in Women Survivors of Intimate Partner Violence**

Inmaculada Garrido León^1^, Maripaz García-Navas Menchero^1^, Ana Isabel de Luis Ruiz^1^, María Soledad Martínez Fernández^1^, Julia Daugherty^1^, Inmaculada Teva García^1^, Natalia Hidalgo Ruzzante^1^

^1^University of Granada, 18012 Granada, Spain

**Introduction**: Psychoneuroendocrinoimmunological (PNEI) diseases are medical conditions explained through the interaction of psychological and neuropsychological processes, the nervous, endocrine, and immune systems. Rather than a fixed list of disorders, PNEI provides a framework for understanding chronic and complex conditions arising from these interactions. These diseases are highly prevalent among women who have experienced Intimate Partner Violence Against Women (IPVAW). Studies report high rates of fibromyalgia, chronic pain, and autoimmune diseases in survivors, affecting 70–95% of them. Survivors also face higher risks of cervical cancer (1.47 to 4.28 times greater), cardiovascular diseases such as hypertension or stroke (33% higher risk), and diabetes (11–46% increased probability). This study analyzed the prevalence of PNEI-related diseases in survivors of IPVAW compared to a control group. **Methodology**: The sample included 165 survivors and 82 controls, both completing an online health questionnaire. **Results**: Chi-square analyses revealed significant differences between survivors and controls in gastrointestinal problems: X^2^_(1,238)_ = 6.833, *p* = 0.009; autoimmune diseases: X^2^_(1,247)_ = 10.563, *p* = 0.001; fibromyalgia: X^2^_(1,238)_ = 11.477, *p* = 0.865. **Conclusions**: Overall, findings align with previous literature, highlighting a specific profile of health problems among survivors of IPVAW. This contributes to advancing research on the long-term medical consequences of such violence and on psychoneuroendocrinoimmunological diseases.

## 2. Oral Communications

**O1. Novel Translational Strategies to Elucidate Neuroimmune Mechanisms in Psychiatric Disorders**

Albert Giralt^1,2,3,4^

^1^Departament de Biomedicina, Facultat de Medicina, Institut de Neurociències, Universitat de Barcelona, 08036 Barcelona, Spain

^2^Institut d’Investigacions Biomèdiques August Pi i Sunyer (IDIBAPS), 08036 Barcelona, Spain

^3^Centro de Investigación Biomédica en Red Sobre Enfermedades Neurodegenerativas (CIBERNED), 28031 Madrid, Spain

^4^Production and Validation Centre of Advanced Therapies (Creatio), Faculty of Medicine and Health Science, University of Barcelona, 08036 Barcelona, Spain

**Introduction**: Psychiatric disorders are complex and multifactorial, arising from both genetic and environmental factors. These conditions present a wide range of symptoms that can be challenging to model and assess in animal studies. In the case of schizophrenia and major depression, recent research has highlighted potential mechanisms that may play crucial roles in its pathophysiology, specifically the miscommunication between the immune system and neuronal circuits. **Methodology**: In our laboratory, we transferred human material such as the secretome of circulating immune cells (or PBMCs) to *in vivo* and *in vitro* models to mimic and study the miscommunication between the central nervous and immune systems in the context of schizophrenia and major depression. After confirming the safety of this methodology and validating several potential underlying molecular mechanisms, we believe that the insights gained from these innovative models will significantly advance our understanding of this altered communication between the two systems in the context of both, schizophrenia and major depression.

**O2. Understanding the Pathomechanism of Endometriosis Through Psychoneuroimmunology**

Orsolya Bustya^1^

^1^Distrito Sanitario Granada-Metropolitano, 18013 Granada, Spain

**Introduction**: Endometriosis is a gynecological disease with chronic pelvic inflammation, which is becoming more and more common nowadays. The etiology of endometriosis may be genetic, hormonal, immunological, nutritional, psychosocial, but these individually provide only partial answers to the understanding of the pathogenesis. Psychoneuroimmunology helps us to understand how emotions, through the limbic and hypothalamo-pituitary systems, affect the endocrine glands and the immune system, and thus the development and progression of endometriosis, taking into account the role of environment and personality traits in its development. The role of women in society has changed significantly over the last half century, both in terms of career development and childbearing, which has increased their exposure to stressors and the effects of estrogen. Psychosocial stress leads to elevated cortisol levels through the hypothalamic-pituitary-adrenal axis and, if sustained, leads to immunosuppression, reduced T-killer lymphocyte activity and increased angiogenesis, which contributes to the development of endometriosis and the growth of these lesions. **Methodology**: This prospective study included 150 women with endometriosis (mean age 35.61) and 72 women without the disease (mean age 35.13), who served as the control group. The study was carried out in the County Emergency Clinical Hospital, Târgu Mureș (Romania). Participants were asked to complete a composite questionnaire that included the Perceived Stress Scale, an Effort-Reward Imbalance Questionnaire and a scale measuring loneliness and social isolation (UCLA Loneliness Scale), in order to assess the extent of personal and work related stress in their lives. **Results**: The results revealed a statistically significant difference, with women with endometriosis reporting higher levels of stress (*p* < 0.0001) and loneliness (*p* = 0.0009) compared to the control group. **Conclusion**: Endometriosis, in terms of its origin, is a multifactorial disease in which the consideration of psychosocial factors may be of great help in the diagnosis, prevention and selection of appropriate treatment.

**O3. Yoga-Induced HPA-Axis Modulation and Vagal Enhancement: Implications for Stress and Emotional Well-Being**

Orsolya Bustya^1^

^1^Distrito Sanitario Granada-Metropolitano, 18013 Granada, Spain

**Introduction**: In modern society, elevated chronic stress underlies many illnesses. Prolonged stress results in allostatic load — the ‘wear and tear’ on the body from sustained physiological adaptation to stressors. This process reflects dysregulation of the autonomic nervous system (ANS) and can lead to cumulative biological consequences involving changes in the nervous, endocrine, and immune systems. Mind-body interventions like yoga can decrease sympathetic tone and increase parasympathetic tone, directly stimulating the vagus nerve and thus returning to homeostasis. Yoga’s main ways to increase parasympathetic tone is through releasing muscle tension, breathing techniques and mindfulness practices. Heart rate variability (HRV) is a biomarker of ANS functioning and is able to assess psychological stress and emotional regulation. Increased vagal activity - reflected in higher HRV - can inhibit excessive hypothalamic-pituitary-adrenal (HPA) axis activation, thereby reducing cortisol secretion and the physiological effects of stress. Conversely, reduced HRV is linked to prolonged HPA axis activation and higher cortisol levels. **Methodology**: Thirteen healthy female participants (mean age: 30 years) were enrolled in a 10-week Hatha Yoga intervention, practicing for one hour 3 times per week. The assessments were conducted at baseline and after completion of the program. Physiological measures included resting heart rate, heart rate response to deep inhalation, and blood pressure. Psychological assessment of emotional balance was assessed using a standardized psychological test. **Results**: The parameters of the yoga participants showed a significant improvement in emotional balance (*p* = 0.047) and enhanced autonomic regulation, reflected by increased HRV (*p* = 0.005). Also, reductions in resting heart rate and blood pressure suggested decreased sympathetic dominance and improved cardiovascular function. These physiological changes imply a downregulation of HPA axis activity. **Conclusion**: The results highlight yoga’s efficacy as an intervention that enhances vagal tone, modulates HPA axis activity, and promotes emotional and physiological resilience to chronic stress.

**O4. The Uterus-Brain Axis and Premenstrual Disorders**

Juana Lafaja Mazuecos^1^, Noelia Serrano Gadea^2^, Elena López Magoñil^2^

^1^NG Clinicas, Integrative Gynecology and Sexology Unit, Doctoral program at the Institute of Bioengineering, Miguel Hernández University, 03202 Elche, Spain

^2^Pain Neuropharmacology and Functional Diversity (NED), Alicante Institute for Health and Biomedical Research (ISABIAL), Research Support Laboratory, Hospital General Universitario Alicante Dr. Balmis, 03010 Alicante, Spain

**Introduction**: Pioneering clinical observations from the 1970s by Dr. Jorge Lolas and Dr. Rodrigo Forés in Chile first suggested a relationship between inflammatory processes in the uterine cervix and profound alterations in mood, cognition, sexual response, and numerous physical symptoms in women during the luteal phase of the menstrual cycle. Today, we still lack a detailed description of the enigmatic uterus-brain axis. While severe cyclical symptoms, experienced by a significant percentage of women, have long been described as premenstrual disorders and Premenstrual Dysphoric Disorder (PMDD), their underlying causes remain poorly understood. A historical gender bias in the study of female genital diseases has limited progress, often focusing exclusively on reproductive aspects and neglecting other areas of women’s health such as mental health and pain. Furthermore, the existence of a specific endometrial microbiota was only recently recognized, challenging the previous assumption of a ‘sterile’ cavity. We can use the established gut-brain axis as a framework for describing a similar axis connecting the uterus to the brain. **Hypothesis**: Our DISFEM research group at Miguel Hernández University of Elche aims to delve deeper into this matter. We hypothesize that a specific phenotype of severe premenstrual disorder and PMDD exists, linked to immunoinflammatory and even autoimmune mechanisms of the reproductive system. The recently described uterus-chemokine-brain axis provides an integrated explanatory model. This model suggests that local uterine inflammation, via the endometrial production of chemokines, is a cause of menstruation-associated symptoms The increased release of chemokines from the uterus is proposed to create an environment of heightened pain sensitivity and neuroinflammation, which is responsible for the psychiatric and cognitive symptoms reported by women, especially during the ovulatory and luteal phases. **Conclusion**: We propose a paradigm shift in our understanding of uterine function. Beyond its reproductive role, the uterus directly impacts the central nervous system. Its inflammation can lead to severe, cyclical systemic symptoms. This new perspective frames the reproductive system as an independent entity with unique characteristics that demand a differential approach, while recognizing its interconnectedness with the gut-brain axis and other body systems.

**O5. Maternal Separation During Infancy Confers Resilience And Neuroimmune Protection To Adult Stress in Females: A Possible Role Of Hippocampal Neurogenesis, Microglia Status And Inflammatory Profile**

Jose Munoz-Martin^1,2^, Patricia Chaves-Peña^1^, María Inmaculada Infantes-López^1,2^, Emma Zambrana-Infantes^3^, Andrea Nieto-Quero^3^, Víctor Martín-Aguiar^3^, Alejandro Zea-Doña^1^, Virginia Carayol-Gordillo^1^, Cristina Ramírez^3^, Margarita Pérez-Martín^1,2^, Carmen Pedraza^2,3^

^1^Departamento de Biología Celular, Genética y Fisiología, Universidad de Málaga, 29010 Málaga, Spain

^2^Instituto de Investigación Biomédica de Málaga y Plataforma en Nanomedicina IBIMA Plataforma BIONAND, 29010 Málaga, Spain

^3^Departamento de Psicobiología y Metodología de las Ciencias del Comportamiento, Universidad de Málaga, 29010 Málaga, Spain

**Introduction**: Maternal separation is an early-life adversity that can cause long-term brain and behavioral changes, increasing vulnerability to stress-related disorders later in life. This study aimed to investigate sex-specific responses to adult stress in mice exposed to early adversity. **Methodology**: Female and male C57BL/6J mice underwent 3-hour daily maternal separation (MS) for 21 consecutive days. At day 60, they underwent a single 2-hour restriction stress (RS) and 24-hours later, they were evaluated in their behavior, hippocampal neurogenesis, microglia and inflammatory status. The experimental groups included Control, RS, MS, and MS+RS. **Results**: Behaviorally, MS+RS females exhibited a reduction in maladaptive stresscoping behaviors: increased motivation to build a nest, reduced hyperactivity in Open Field Test and increased swimming in the Forced Swimming Test compared to Control or MS females. In a cellular basis, MS+RS females presented increased number of ramified late-stages DCX+ cells in the ventral hippocampus suggesting enhanced maturation of newborn granular neurons. This change in ventral hippocampal neurogenesis might have a role in resilience and antidepressant like behaviors as evidenced by the behavioral results. Furthermore, microglia morphology of MS+RS females in the ventral hippocampus showed a decreased cell body size and increased circularity with a sparsely distribution (increased separation between each pair of cells). The cytokine profile of MS+RS and MS females gravitated toward an anti-inflammatory status with increased levels of hippocampal IL-4, highly linked to microglial neuroprotection and neuronal survival, and decreased levels of IL-1β (pro-inflammatory), suggesting a possible neuroimmune mechanism for a long-term protection of female hippocampi. On the contrary, MS+RS males did not display a clear stress-reactive pattern in either behavior or cellular changes, with only RS males showing anxious behavior in Open Field Test. **Conclusions**: This female-specific results provide insights into the neurobiological basis of susceptibility or resilience to disorders such as anxiety and depression.

**O6. The Role of APOAI in Translational Studies Addressing Alcohol Abuse, Inflammation and Cognitive Impairment**

Laura Orio^1,2,3^, Berta Escudero^1,2,3^, Leticia López-Valencia^1,2,3^

^1^Department of Psychobiology, Complutesnse University of Madrid, 28040 Madrid, Spain

^2^Instituto de Investigación Sanitaria Hospital Universitario 12 de Octubre (imas12), 28041 Madrid, Spain

^3^Riapad: Research Network in Primary Care in Addictions, Spain

**Introduction**: Bacterial lipopolysaccharide (LPS) is translocated from the gut to the blood after alcohol abuse and activates the immune system with consequences in neuroinflammation and cognition. Apolipoproteins are compounds altered by alcohol with high affinity to LPS which may be involved in its transport and/or elimination and recently linked to cognition. Aim: We explore alterations in several apolipoproteins in rat and mice models of alcohol abuse and in humans with alcohol use disorder (AUD) and studied associations with inflammation and cognitive decline. **Methodology**: Intragastric administrations of alcohol in binges were used for rats, mice were exposed to a mixed two-bottle choice-CIE vapor exposure paradigm and human AUD patients were recruited according to DSM-5 diagnosis. Apolipoproteins and inflammatory markers were measured by ELISA, Multiplex assays or immunohistochemistry, apolipoprotein-LPS aggregates and immune TLR4 receptors were measured by western blot and co-immunoprocipitation. Cognitive deterioration and memory impairments were assessed by TEDCA test and Wechler Memory Scale-IV in humans and OLT/NOR test in animals. **Results**: Several apolipoproteins were altered in rat/mice models of alcohol abuse/dependence and in AUD patients, being plasma APOAI consistently upregulated in animals and humans. APOAI form aggregates with parts of LPS in the rat female brain after alcohol exposure. Plasma APOAI correlated with inflammation and general cognitive impairment in AUD patients and, specifically, with poor memory in mice and humans. **Conclusion**: The upregulation of plasma APOAI after alcohol abuse and dependence in animals and humans and the use of APOAI mimetic peptides will be discussed in the context of alcohol-induced neuroinflammation and cognitive impairment.

**O7. Impact of Cocaine and Ethanol Co-Use on Blood–Brain Barrier Integrity: Insights From *In Vivo* and *In Vitro* Approaches**

Lucía Garrido-Matilla^1^, Carlos Vera-Fernández^1^, Eliane Swely Sanches^2,3,4,5^, Daniela Simões^2,3,4,5^, Alba Arranz ^1^, Alberto Marcos^1^, Emilio Ambrosio^1,†^, Ana Paula Silva^2,3,4,5,†^

^1^Psychobiology Department, School of Psychology, National University for Distance Learning (UNED), 28040 Madrid, Spain

^2^Institute of Pharmacology and Experimental Therapeutics, Faculty of Medicine, University Coimbra, 3000-548 Coimbra, Portugal

^3^Institute for Clinical and Biomedical Research (iCBR), Faculty of Medicine, University Coimbra, 3000-548 Coimbra, Portugal

^4^Center for Innovative Biomedicine and Biotechnology (CIBB), University Coimbra, 3004-504 Coimbra, Portugal

^5^Clinical Academic Center of Coimbra (CACC), 3004-504 Coimbra, Portugal

^†^These authors contributed equally.

**Introduction**: Drug addiction is a major public health problem, with the polysubstance use of cocaine and ethanol representing one of the most prevalent patterns. It is a chronic relapsing disorder characterized by compulsive drug-seeking behaviour and neurobiological adaptations, including neuroinflammation, which is known to strongly affect the blood–brain barrier (BBB). However, the effects of combined cocaine and ethanol exposure on the BBB remain unclear. In this context, the pro-inflammatory cytokine IL-17A is of particular interest, as it has been linked to BBB disruption. Moreover, our group has reported elevated IL-17A levels in rats reinstating cocaine-seeking behaviour, suggesting a potential role in both barrier integrity and relapse vulnerability. **Methodology**: In this study, we investigated the impact of combined cocaine and ethanol administration on the BBB and explored the potential involvement of IL-17A signalling. We employed a dual approach: (1) A rat model of cocaine plus ethanol-seeking behaviour, analysing gene expression of tight and adherens junction proteins in addiction-related brain regions, as well as immune markers in the spleen. (2) An *in vitro* human BBB model composed of brain endothelial cells (hCMEC/D3), assessing barrier integrity upon exposure to cocaine and ethanol, and examining junction proteins and IL-17A receptors. **Results**: In animal studies, analysis of the hippocampus and striatum revealed a sex-dependent reduction in claudin-5 and VE-cadherin levels following cocaine plus ethanol self-administration and withdrawal. Increased expression of IL-17 receptors, particularly IL-17 receptor C, was also observed. *In vitro*, both co-exposure and single-substance exposure reduced transendothelial electrical resistance, indicating compromised BBB integrity. BBB disruption was associated with decreased claudin-5 levels and a positive trend in IL-17 receptor C expression. Gene expression analysis from the spleen is ongoing. **Conclusions**: These preliminary results suggest that cocaine and ethanol co-exposure induces a sex-dimorphic pattern of BBB disruption, likely through alterations in intercellular junctions and IL-17A signalling.

**O8. Early Detection of Peripheral Alzheimer’s Disease Biomarkers that Reflects The Neuroimmune Status of the Hippocampus**

Isabel Moreno-Madrid^1,2^, D.D. Molina-Sánchez^1^, Amelia Díaz-Casares^1,2^, P. Viñas-Morales^1^, J.C. Arrabal-Gómez^1,3,4^, E. Díaz-Sánchez^1,3^, M.A. Barbancho-Fernández^1,2^, J.A. Sánchez-Pérez^1,2,5^, E. Blanco-Reina^1,2^, R. Beltrán-Casanueva^6,7^, J.V. Bayolo-Guanche^1,6^, M. Tome-Garcia^1,2,8^, L. Carazo-Barrios^1,9^, J.A. Reyes-Bueno^1,4^, C. Pérez-Enriquez^1^, J. Romero-Imbroda^1,4,10^, K. Fuxe^7^, D.O. Borroto-Escuela^2,6^, N. García-Casares^1,2^, P. Serrano-Castro^1,2,3,4^, M. Narváez-Peláez^1,2,3^

^1^ImbrainLab-Cátedra Imbrain, Facultad de Medicina, Universidad de Málaga, 29010 Málaga, Spain

^2^Instituto de Investigación Biomédica de Málaga, Universidad De Málaga, 29010 Málaga, Spain

^3^Vithas Málaga, Grupo Hospitalario Vithas, 29010 Málaga, Spain

^4^Unit of Neurology, Hospital Regional Universitario de Málaga, 29010 Málaga, Spain

^5^Unit of Psychiatry, Hospital Universitario Virgen de la Victoria, Universidad De Málaga, 29010 Málaga, Spain

^6^Receptomics & Braindisorders Lab, Facultad de Medicina, Universidad De Málaga, 29010 Málaga, Spain

^7^Department of Neuroscience, Karolinska Institutet, SE-171 65 Stockholm, Sweden

^8^Unit of Endocrinology, Hospital Regional Universitario de Málaga, 29000 Málaga, Spain

^9^Unit of Neurology , Hospital Universitario de Jaén, 23007 Jaén, Spain

^10^Quirón Salud Málaga, Grupo Hospitalario Quirón, 29004 Málaga, Spain

**Introduction**: The neuroimmunological changes associated with Alzheimer’s disease (AD) begin to appear long before symptoms manifest. In previous research on its treatment, we found improvements in spatial memory associated with an increase in the formation of NPY1R heteroreceptor complexes with GALR2 and/or TrkB and an improvement in neurogenesis and plasticity in the dentate gyrus of the hippocampus in physiological models. Based on this, the present research sought to address the early detection of AD through the observation in hippocampal cell samples and white blood cells of NPY1R-GALR2 and NPY1R-TrkB complexes as possible early biomarkers of the disease, using the *in-situ* PLA technique. **Methodology**: An AcellsiRNA model for NPY1R inoculated intracerebroventricularly was developed in rats to render this receptor inactive, imitating this alteration in AD. Spatial memory was assessed after eight days using the object-inplace test, neurogenesis was analysed using the doublecortin (DCX) marker in the dentate gyrus of the dorsal hippocampus, and *in situ* PLA tests were performed in the samples. **Results**: The results show a decrease in brain tissue and white blood cells in the formation of heteroreceptor complexes with NPY1R, but no alteration was observed in the number of DCX-labelled cells or in spatial memory. These results are compared with a bilateral olfactory bulbectomy model, which reproduces an AD model in rats. In this model there is an alteration in neurogenesis in the dentate gyrus of the hippocampus associated with a decrease in the formation of NPY1R heteroreceptor complexes and a decrease in spatial memory. **Conclusions**: We suggest that it is possible to detect changes in NPY1R-GALR2 and NPY1R-TrkB complexes in blood cells before the onset of symptoms, making this a potential early biomarker of the disease. Further investigation is required to confirm this statements.

**O9. Allostatic Load And Risk of Developing Cancer in the EPIC-Granada Cohort**

Dafina Petrova^1,2,3,4^, José María Gálvez-Navas^1,2,3^, Daniel Redondo-Sánchez^1,2,3^, Blanca Madrid Pérez-Esparza^1,2^, Laura León^1^, Encarnación González-Flores^1,4^, María del Señor López-Vélez^1,4^, Alicia Rubio Pilares^1,5^, María-José Sánchez^1,2,3^

^1^Instituto de Investigación Biosanitaria ibs.GRANADA, 18012 Granada, Spain

^2^Escuela Andaluza de Salud Pública, 18010 Granada, España

^3^CIBER de Epidemiología y Salud Pública (CIBERESP), 28029 Madrid, Spain

^4^Medical Oncology, Hospital Universitario Virgen de las Nieves, 18014 Granada, Spain

^5^Universidad de Granada, 18071 Granada, Spain

**Introduction**: Chronic stress has long been suspected to increase the risk of cancer. Basic and preclinical research has identified multiple molecular and systemic mechanisms through which chronic stress can influence cancer progression. However, epidemiological evidence from prospective cohort studies using questionnaire-based measures of chronic stress remains inconclusive. The concept of allostatic load, which captures the cumulative biological burden of chronic stress, provides an alternative approach to assessing stress exposure in relation to cancer. **Methodology**: We conducted a case-control study embedded in the European Prospective Investigation into Cancer and Nutrition (EPIC)-Granada cohort recruited 1992-1996. Participants (n = 7879) completed multiple lifestyle questionnaires, underwent physical examination, and donated blood samples. The 964 incident cancer cases that occurred until 2018 were matched to 964 controls on age, sex, fasting status, and time since blood draw. An allostatic load index was calculated using a distributional algorithm from 12 neuroendocrine, cardiometabolic, and immune biomarkers determined in serum samples and supplemented with diagnostic and medication information. **Results**: On average, 15 years had elapsed between the blood draw and the cancer diagnosis of cases. Cases had a significantly higher pre-diagnostic allostatic load than controls (Mean (standard deviation):7.6 (3.2) vs. 6.9 (3.0), respectively). In multiple conditional logistic regression, higher allostatic load was associated with higher overall cancer risk, with OR = 1.07 (95% CI 1.04–1.11). By tumor type, allostatic load was associated with higher risk specifically for breast (OR = 1.13, 1.04–1.23), colorectal (OR = 1.13, 1.02–1.25), and female reproductive system (OR = 1.21, 1.06–1.39) cancer. The allostatic load biomarkers showing significant differences (*p* < 0.05) between cases and control included cortisol, DHEA-S, IGF-1, HOMA-IR, albumin, and total cholesterol. **Conclusions**: Higher pre-diagnostic allostatic load was associated with higher cancer risk. Allostatic load can help bridge the gap between basic and epidemiological research on stress and cancer, contributing towards personalized prevention strategies.

**O10. Neuroimmunomodulation, the Importance of Combined Therapies to Stimulate Resoleomics and Modulate Neuroimmunometabolic Programming**

Paola Amaya^1^, Ana Rivas^1^

^1^Medicina privada, Bogotá, Colombia

**Introduction**: Psychoneuroimmunology (PNI) has demonstrated the interaction between the nervous, immune, and endocrine systems, highlighting their role in regulating physical and mental health. Within this framework, neuroimmunomodulation emerges as a key approach in the treatment of chronic diseases by integrating autonomic, inflammatory, and metabolic responses, which are fundamental for the organism’s adaptation. **Methodology**: A literature review was conducted in PubMed, Scopus, and Web of Science (2009–2024), prioritizing studies on neuroimmunomodulation, the autonomic nervous system (ANS), vagus nerve, resolvomics, microbiota, and multimodal therapies. **Results**: The ANS acts as a master regulator of neuroimmunometabolic homeostasis. Mild autonomic dysfunction precedes multiple chronic diseases, and its assessment through heart rate variability (HRV) is a relevant clinical biomarker. The vagus nerve, with its nucleus of the solitary tract—the main afferent center receiving visceral information—and its efferent nuclei, the dorsal motor nucleus of the vagus and the nucleus ambiguus, is a fundamental center of vegetative control. It has great potential to modulate inflammation through cholinergic anti-inflammatory pathways. The gut microbiota represents a central axis in immune and metabolic programming. Multimodal therapies show efficacy in restoring autonomic flexibility, stimulating inflammatory resolution, and enhancing the organism’s ability to maintain homeostasis. **Conclusions**: A multimodal therapeutic approach actively stimulates endogenous mechanisms of inflammatory resolution and may contribute to the restoration of a balanced immune profile. Strategies may be simple, such as optimizing sleep patterns, circadian exposure to sunlight, daily physical activity, or breathing exercises. They may also be more complex, including gut microbiota interventions, optimization of hepatic detoxification processes, specific nutritional support, or psycho-emotional interventions such as mindfulness, cognitive-behavioral therapy, emotional regulation techniques, and advanced processes of neurostimulation or immunomodulation.

**O11. Loneliness, Immune Cells and Psychosocial Intervention: Indirect Effects of Emotional Loneliness On Monocytes in Older Adults**

Juan Ignacio Grec^1^, Leyre Castillejo Sanz^1^, María Roman Moren^1^, Raúl Ballesta Barrera^1^, Sara García Herranz^1^, María del Carmen Díaz Mardomingo^1^, Carmen Donaire Saz^1^, Cynthia Diaz-Silveira^2^, Adrián Galiana Rodríguez^3^, Shishir Baliyan^1^, César Venero Núñez^1^

^1^Department of Psychobiology, National University of Distance Education (UNED), 28040 Madrid, Spain

^2^Department of Psychology, Faculty of Health Sciences, Universidad Rey Juan Carlos, 28922 Madrid, Spain

^3^Department of Psychology, Faculty of Health Sciences and Education, Universidad a Distancia de Madrid (UDIMA), 28400 Madrid, Spain

**Introduction**: Loneliness has been linked to dysregulation of the immune system in aging, including alterations in inflammatory processes and immune cell activity in aging. Psychosocial interventions are a promising approach to alleviate loneliness and potentially modulate its psychobiological consequences. **Methodology**: We investigated the effects of a 20-session psychosocial intervention program designed to reduce loneliness in a sample of 50 community-dwelling older adults. The program included modules on social skills, self-esteem and personal control, intergenerational exchange, life-story sharing, and mindfulness practices. Pre–post intervention assessments included social and family loneliness (Social and Emotional Loneliness Scale for Adults-SESLA) and immune markers such as monocytes, lymphocytes, fibrinogen, homocysteine, and C-reactive protein. Regression-based mediation analyses (PROCESS) were conducted, adjusting for age, sex, education level and depressive symptoms as measured by the Geriatric Depression Scale- GDS. **Results**: Results showed that participation in the intervention program significantly reduced social loneliness. Moreover, mediation analysis revealed a significant indirect effect of the intervention on the change in monocyte levels through reductions in family loneliness. Specifically, participants in the intervention group showed greater decreases in family loneliness (β = –0.579, *p* = 0.041), which in turn predicted reductions in monocytes (β = 0.327, *p* = 0.023). The indirect pathway was statistically significant (β = –0.1899, 95% CI [–0.4518, –0.0014]). The direct effect of the intervention on monocytes was not significant (β = 0.435, *p* = 0.22), highlighting the mediating role of family loneliness in explaining the observed biological changes. **Conclusions**: These findings provide novel evidence that psychosocial interventions not only alleviate perceived loneliness but may also influence immune function in older adults. By identifying family loneliness as a key mediator, the study suggests a specific pathway linking psychosocial well-being to inflammatory regulation. Further research is warranted to examine to examine long-term effects and to expand biomarker profiling.

**O12. Environmental Causes of Disease in the Anthropocene, a Paramount PNEI View**

Mauro Bologna^1^

^1^Department of life, Health and Environmental Sciences, General Pathology, University of L’Aquila Medical School, 67100 L’AQUILA, Italy

We (the humans) live in a terrestrial environment, from which we receive air, food, water and all the resources for our needs. Each individual engages a daily and intimate relationship with the environment, where we must consider that we are not owners but guests, in a reciprocal interaction with all other forms of life. One World (in the sense that we all live on the same planet) and One Health (in the sense that all live beings on the planet share many health and disease interactions) are therefore the basic phylosophical, biomedical and practical paradigms of ecology on Earth: the common house for all live beings, including us, on such planet (the only one available for the known ecosystems so far). We received terrestrial resources from our ancestors (and the ecological cohabitants), through our parents, and we leave what remains to our children and descendants who should hopefully be able to live on Earth in future times with the same chances and quality of life that was offered to us. The environment however accumulates the consequences of all the preceding insults caused by us and by our human predecessors, who started centuries and centuries ago to extract, construct, modify, pollute every part of the planet, which now carries all the human modifications evident in the present times (Anthropocene). Since the industrial revolution (a little more than two centuries ago) humans potentiated enormously their capacity to modify the environment, often for improving human life, but almost always by destroying ecosystems and depauperating the natural resources in all aspects. The PNEI paradigm that inspires us, a group of physicians and health professionals of the XXIst century, and also human beings able to recognize and appreciate the mind-body relationships within each individual, may be very useful to interpret the complex interplay network equilibria existing in ecological systems between different forms of life and regulating health and disease in every living organism. And such a network-based PNEI paradigm should be the best basic knowledge to appreciate and correct the environmental causes of disease, before it is too late. As for climate on Earth in the XXIst century, many observations show us that it may be already too late to make changes to revert to sustainable equilibria. We shall discuss here some peculiar aspects of these complex but fundamental issues regulating ecosystems and human Heath, with the PNEI paradigm well in perspective.

## 3. Poster Communications

**P1. Oxytocin Reactivity by Basal Levels in Intimate Partner Violence Offenders: Links to Socioemotional Regulation**

Andrea Antonio Gheorghe^1^, Javier Comes-Fayos^2^, Raquel Marquino Fernández^1^, Marisol Lila-Murillo^3^, Ángel Romero-Martínez^1^, Luis Moya-Albiol^1^

^1^Department of Psychobiology, University of Valencia, 46010 Valencia, Spain

^2^Faculty of Health Sciences, Valencian International University, 46002 Valencia, Spain

^3^Department of Social Psychology, University of Valencia, 46010 Valencia, Spain

**Introduction**: Emerging evidence suggests oxytocinergic dysregulation in intimate partner violence (IPV) offenders and links altered oxytocin (OXT) function with risk factors for violent behavior. Because OXT effects are context-dependent and shaped by the salience of social signals, reactivity to social stimuli provides a dynamic index of system functioning. Traumatic brain injury (TBI) may further impact these pathways. To examine whether basal OXT clusters differ in oxytocin reactivity (ROXT) to an empathic audiovisual induction task and to assess the impact of TBI. **Methodology**: IPV offenders (n = 32) were classified into High OXT (n = 9) and Low OXT (n = 23) clusters based on log-transformed basal OXT. ROXT was computed as area under the curve with respect to increase (AUCi) during an empathic audiovisual induction task involving viewing people suffering violence. TBI potential presence was coded (Yes = 8, No = 24). A between-subjects univariate ANOVA tested Cluster and TBI effects on ROXT. **Results**: The overall model was significant (F_(2,29)_ = 11.271, *p* < 0.001), explaining 43.7% of the variance in ROXT (R^2^ = 0.437, adj. R^2^ = 0.399). A main effect of Cluster emerged: Low OXT showed lower reactivity than High OXT (F_(1,29)_ = 21.925, *p* < 0.001, η^2^_p_ = 0.431). TBI was not significant (F_(1,29)_ = 0.357, *p* = 0.555). **Conclusions**: A low basal OXT cluster is associated with attenuated ROXT, delineating a hyporeactive phenotype (low baseline + small increase) independent of TBI. This pattern may reflect reduced affiliative activation and stress buffering in response to social cues, with consequences for socioemotional regulation during couple conflict. Interventions that enhance the salience of safe social signals through targeted training and context design may constitute an important protective factor to optimize social cognition in offenders, both with and without TBI.

**P2. Sex, Menstrual Cycle, and Hormonal Contraceptives Effects on Endogenous and Exogenous Attention**

Bernal Antonio^1,2^, Aragonés Lara^2^, Paolieri Daniela^2,3^

^1^Department of Psychobiology, University of Granada, 18071 Granada, Spain

^2^Mind, Brain and Behavior Research Center (CIMCYCI), 18071 Granada, Spain

^3^Department of Experimental Psychology, University of Granada, 18071 Granada, Spain

**Introduction**: Attention can be oriented to spatial locations in two distinct ways: endogenously (driven by our goals, intentions, or task demands) or exogenously (in response to salient or potentially relevant stimuli). Both forms of attention engage a bilateral fronto-parietal network, with a high density of receptors for gonadal hormones. Therefore, the present study investigates sex differences, as well as the effects of the menstrual cycle and hormonal contraceptive use, on these attentional processes. **Methodology**: The study involved 21 men, 64 women with a natural menstrual cycle (21 participated during the early follicular phase, 22 during the ovulatory phase, and 21 during the mid-luteal phase), and 23 women using hormonal contraceptives, who performed the task during the active hormonal phase. After collecting saliva samples to assess gonadal hormone levels, participants completed both an endogenous and exogenous attention orienting task, each containing valid, invalid, and neutral trials. Inverse efficiency (accuracy/response time) was used as the performance measure. **Results**: Testosterone levels were higher in the male group, estradiol levels were elevated in the ovulatory group, and progesterone levels were higher in the luteal group. The ovulatory group demonstrated significantly greater inverse efficiency during invalid trials of the exogenous condition compared to men, the luteal group, and hormonal contraceptive users. No other significant differences were observed across the groups. **Conclusions**: These results suggest that women in the ovulatory phase experience greater difficulties in comparison with the other groups in disengaging attention and inhibiting irrelevant information.

**P3. Hair Cortisol Concentration in Male Perpetrators of Intimate Partner Violence Against Women and its Relationship With Sociodemographic and Sentence-Related Variables**

María Ángeles García-León^1^, Natalia Bueso-Izquierdo^2,3^, María Isabel Peralta-Ramírez^3,4^, Raquel González-Pérez^5^, Miguel Pérez-García^3,4^

^1^Department of Personality, Assessment, and Psychological Treatments, Universidad de Sevilla, 41018 Seville, Spain

^2^Department of Developmental and Educational Psychology, University of Granada, 18071 Granada, Spain

^3^Mind, Brain and Behavior Research Center (CIMCYC), University of Granada, 18071 Granada, Spain

^4^Department of Personality, Evaluation and Psychological Treatment, University of Granada, 18071 Granada, Spain

^5^Department of Pharmacology, CIBERehd, School of Pharmacy, University of Granada, 18071 Granada, Spain

**Introduction**: To understand the violent behavior of perpetrators of intimate partner violence against women (IPVAW), the profile of convicted men is currently being studied from a multiglobal perspective, including the biological dimension. A literature review shows that the biological markers of IPV perpetration play a significant role in the etiology of this specific type of violence. Neuropsychology, neuroimaging and psychophysiology studies have been conducted to understand these men’s violent acts, yet few have addressed the role of hormones in the violent behavior of this population. Among all the hormones involved in violent behavior, cortisol and testosterone have been found to be the most significant. **Methodology**: A total of 627 male volunteers convicted of IPVAW participated in the study and completed a semi-structured interview covering sociodemographic information, conviction-related variables, health and life habits, and childhood experiences. Hair samples were collected and hair cortisol concentrations were analyzed. **Results**: Compared to a Spanish reference population IPVAW perpetrators were predominantly in the highest and lowest percentiles of HCC distribution. While most sociodemographic and life habit variables were unrelated to HCC, non-Spanish nationality and childhood physical abuse were associated with higher cortisol levels. Additionally, significant associations were found between HCC and crime-related factors, such as conviction length, type of charge, and unfair charge perceptions. Specifically, shorter and intermediate conviction lengths, multiple partner charges, and acknowledgment of physical abuse charges were linked to increased HCC. **Conclusions**: These findings highlight the importance of integrating psychological and biological factors to understand IPVAW perpetration.

**P4. Association Between Vitamin D and Cognitive Performance in Spanish Adults**

Mario Tomé-Fernández^1^, Marcelo Saval-Calvo^2^, Miriam Sánchez-Sansegundo^1,3^, Ana Zaragoza-Martí^3,4^, Jorge Azorín-Lopez^2^

^1^Department of Health Psychology, Faculty of Health Science, University of Alicante, 03690 Alicante, Spain

^2^Department of Computer Science and Technology, University of Alicante, San Vicente del Raspeig, 03690 Alicante, Spain

^3^Alicante Health and Biomedical Research Institute, ISABIAL Foundation. 03010 Alicante, Spain

^4^Department of Nursing, Faculty of Health Science, University of Alicante, 03690 Alicante, Spain

**Introduction**: Several studies have documented associations between specific micronutrient levels and cognitive performance. An adequate intake of vitamins influences neurotransmitter synthesis, neuronal activity, and the structural integrity of cell membranes. The aim of this study was to examine differences in vitamin intake among four groups classified based on cognitive performance. **Methodology**: A cross-sectional study was carried out with a sample of 230 Spanish adults participating in the Tech4Diet-Person project. Dietary intake was assessed using a semiquantitative Food Frequency Questionnaire (FFQ), while cognitive performance was evaluated w computerized neuropsychological battery. Participants were categorized into four groups based on their cognitive performance (very low, low, average, and high), and a one-way ANOVA was conducted to examine differences in micronutrient intake. **Results**: The sample included 230 Spanish adults (M = 45.73, SD = 10.1), 61.6% women. The results revealed statistically significant differences in vitamin D intake (F = 4.623, *p* = 0.004). Specifically, the very low performance group consumed significantly less vitamin D than the high-performance group (8.50 ± 13.33 mg vs. 30.12 ± 27.47 mg, *p* = 0.028, d = 0.95). **Conclusions**: The results reveal a significant association between low vitamin D intake and impaired cognitive performance. Systematic reviews have shown that vitamin D supplementation can lead to improvements in cognitive domains such as memory, attention, and executive function. Moreover, observational and longitudinal studies have found that serum levels below the threshold are associated with global cognitive decline. However, this relationship is not consistent across all cognitive functions or age groups, and causality has yet to be fully established.

**P5. Vitamin Intake as a Predictor of Prefrontal Dysfunction: A Machine Learning Approach**

Mario Tomé-Fernández^1^, Bernabé Sánchez-Sos^2^, Miriam Sánchez-Sansegundo^1,3^, José Antonio Hurtado-Sánchez^3,4^, Andrés Fuster-Guilló^2^

^1^Department of Health Psychology, Faculty of Health Science, University of Alicante, 03690 Alicante, Spain

^2^Department of Computer Science and Technology, University of Alicante, San Vicente del Raspeig, 03690 Alicante, Spain

^3^Alicante Health and Biomedical Research Institute, ISABIAL Foundation, 03010 Alicante, Spain

^4^Department of Nursing, Faculty of Health Science, University of Alicante, 03690 Alicante, Spain

**Introduction**: Vitamin intake is closely related to prefrontal cortex functioning, a brain region essential for higher-order cognitive and behavioral processes. The objective was to analyse the relationship between vitamins and prefrontal dysfunction by comparing three feature selection models. **Methodology**: A cross-sectional study was carried out with a sample of 223 Spanish adults participating in the Tech4Diet-Person project. Dietary intake was assessed using a semiquantitative Food Frequency Questionnaire (FFQ), and prefrontal dysfunction was assessed using the Short Prefrontal Symptoms Inventory (PSI-20). The Python programming language was used with the Machine Learning library: Scitik-Learn and the Random Forest Regressor. The feature selection methods used for comparison were: Recursive Feature Elimination, Drop-Column Importance and Permutation Feature Importance. **Results**: The sample consisted of 223 Spanish adults (M = 45.64, SD = 10.10), of whom 38.05% were men and 61.95% were women. The model that explained the greatest proportion of variance was the Drop-Column Importance model (MAE = 8.06, MSE = 108.66, R^2^ = 0.23, MAPE = 0.72, RMSE = 10.42, adjusted R^2^ = 0.20). The predictors of the total PSI score were the following vitamins: vitamin C, niacin, thiamine, folate, riboflavin, vitamin B6, and vitamin E. **Conclusions**: This study provides evidence that specific vitamins are significant predictors of prefrontal dysfunction in adults. The Drop-Column Importance model demonstrated the strongest explanatory power, underscoring the relevance of nutritional factors in cognitive health. These findings highlight the importance of adequate vitamin intake as a potential protective factor against prefrontal symptoms, reinforcing the role of nutrition in brain function and suggesting avenues for preventive strategies in public health.

**P6. Effects of Sleep Duration on Perceived Stress and Cardiac Autonomic Recovery Under Cognitive-Social Stress**

Carolina Sarrate-Costa^1^, Luis Moya-Albiol^1^, Ángel Romero Martínez^1^

^1^Department of Psychobiology, Faculty of Psychology, University of Valencia, 46010 Valencia, Spain

**Introduction**: Current literature shows that shorter sleep duration (<7 h/night in adults) is linked to increased allostatic load, whereas sufficient sleep helps maintain the autonomic balance. However, evidence is limited on how chronic sleep insufficiency affects autonomic activity, especially during post-stress recovery, under cognitive and social demands. This study quantifies the effects of habitual sleep duration on perceived stress and cardiac autonomic activity (heart rate, HR) at baseline, during a cognitive-social stressor, and in the recovery period. **Methodology**: 63 healthy adults (20–78 years, M = 38.60, SD = 13.86) reported their average nightly sleep. Participants completed neuropsychological tasks in front of two evaluators while HR was continuously recorded through a physiological ambulatory instrument (VU-AMS). Perceived stress was rated on a numeric scale after the stressor. **Results**: Results showed that sleep duration was associated with lower perceived stress (B = –0.461, SE = 0.226, *p* = 0.046). Perceived stress, in turn, was positively related to HR at baseline (B = 1.369, SE = 0.676, *p* = 0.047), during the task (B = 1.472, SE = 0.699, *p* = 0.039), and during recovery (B = 1.383, SE = 0.632, *p* = 0.032). Importantly, there was no statistically significant association between sleep duration and HR at any of the recorded periods. Taken together, these results partially align with allostatic-load models and extend them by indicating a patter consistent with an indirect link between sleep and autonomic activity via increased perceived stress.

**Conclusions**: This study states that higher perceived stress increases and keeps allostatic load elevated for longer, a profile linked to higher cardiovascular risk, immune and endocrine dysregulation, and poorer task performance and decision-making. In this way, is has been empathized the importance of combining sleep-health promotion and stress-management strategies to optimize autonomic adaptation in everyday cognitive-social demands. Future studies should test this indirect pathway longitudinally and manipulate sleep experimentally to determine causal effects on stress and cardiac recovery.

**P7. Does Spiritual Well-being Improve Metabolic Control in Type II Diabetes?**

M.D. Fernández-Pascual^1^, A. Reig-Ferrer^1^, A.Mª. Santos-Ruiz^1^, C. Gisbert-Sellés^2^, C. Sánchez-Botella^2^, J.L. Talavera-Biosca^2^

^1^Department of Health Psychology, Faculty of Health Sciences, University of Alicante, 03690 Alicante, Spain

^2^Primary Care Center San Vicente del Raspeig I, 03690 Alicante, Spain

**Introduction**: Psychological alterations frequently coexist with type II diabetes mellitus (T2DM), potentially impairing functionality, reducing quality of life, and complicating metabolic regulation. The present study aimed to examine the associations between metabolic control, spiritual well-being, and perceived health in patients with uncontrolled T2DM. **Methodology**: A descriptive, cross-sectional, correlational design was applied, including 105 adults diagnosed with uncontrolled T2DM (HbA1c >7%). The sample consisted of 60 men (M = 64.02, SD = 10.53) and 45 women (M = 68.1, SD = 9.47). Clinical and laboratory data were obtained from electronic health records. Spiritual well-being was assessed with the Spanish version of the Meaning in Life Scale (MiLS-Sp), and perceived health was evaluated using the General Health Questionnaire (GHQ-12). All scores were standardized to a 0–10 scale. **Results**: Results indicated moderate mean scores for Peace (M = 6.6), whereas Purpose (M = 4.9), Lack of Meaning (M = 3.4), and Benefits of Spirituality (M = 3.5) were lower. The overall MiLS-Sp score was 4.7. HbA1c was negatively associated with Purpose (r = –0.23, *p* < 0.05) and the total MiLS-Sp score (r = –0.23, *p* < 0.05). Perceived health correlated negatively with Purpose (r = –0.40, *p* < 0.01), Peace (r = –0.50, *p* < 0.01), and the total MiLS-Sp score (r = –0.50, *p* < 0.01), and positively with Lack of Meaning (r = 0.32, *p* < 0.01). The mean GHQ-12 score was 3.03 (SD = 2.85). **Conclusions**: These findings suggest that higher levels of meaning in life are associated with better metabolic control and self-rated health in patients with uncontrolled T2DM. Nevertheless, the cross-sectional design and sample size impose limitations, preventing causal inferences. Longitudinal research is required to clarify the potential protective role of spiritual well-being in diabetes management.

**P8. Neuroendocrine Sex-Related Differences After Acute Social Defeat Stress in Pubertal CD-1 Mice**

Nerea Perez-Arriazu^1^, Alina Diez-Solinska^1^, Oscar Vegas^1,2^, Garikoitz Azkona^1^

^1^Department of Basic Psychological Processes and their Development, University of The Basque Country (UPV/EHU), 20018 Donostia-San Sebastian, Spain

^2^Biogipuzkoa Health Research Institute, 20014 Donostia-San Sebastian, Spain

**Introduction**: Even though women are primarily affected by social stress-related psychiatric disorders, the majority of preclinical research has been done on male mice because there aren’t many trustworthy female social stress animal models. Owing to the different social dynamics between the sexes, models that have been proposed for one sex have proved ineffective for the other. A defeat stress protocol, based on the application of adult male urine, was employed in our investigation. The present study aimed to validate the defeat stress protocol in pubertal mice under an acute paradigm and investigate potential neuroendocrine and immune response sex discrepancies following the defeat stress protocol. **Methodology**: The social defeat protocol was applied to male and female adolescents in three sessions over one day. **Results**: The behavioural and corticosterone results indicated that the model worked as intended in the laboratory. The endocrine results showed that progesterone and testosterone levels were lower in stressed females than in non-stressed ones, but no differences were observed between male groups. No differences in hypothalamic IL-6 and TNF-α were observed. **Conclusions**: Overall, the results of this study highlight the necessity of doing preclinical research with equal consideration for both sexes.

**P9. Hormonal Ratios in Intimate Partner Violence Perpetrators: Associations With Emotional Decoding And Alexithymia**

Raquel Marquino Fernández^1^, Javier Comes-Fayos^2^, Antonio Gheorghe^1^, Marisol Lila^3^, Ángel Romero-Martínez^1^, Luis Moya-Albiol^1^

^1^Department of Psychobiology, University of Valencia, 46010 Valencia, Spain

^2^Faculty of Health Sciences, Valencian International University, 46002 Valencia, Spain

^3^Department of Social Psychology, University of Valencia, 46010 Valencia, Spain

**Introduction**: Socio-affective deficits, such as poor emotional decoding and high alexithymia, are significant risk factors in intimate partner violence perpetrators (IPVp). Evidence links these deficits to altered hormone levels involved in empathy (oxytocin, OXT), dominance (testosterone, T), and stress response (cortisol, C). Further research is needed to clarify hormone interactions and their impact on socio-affective functions. This study examined differences between IPVp and a control group (CG) in emotional decoding, alexithymia, and affective response during an empathy-induction task, and explored associations with hormonal ratios OXT/T, OXT/C, and T/C. **Methodology**: Groups (IPVp, n = 12, CG, n = 12) completed the Reading the Mind in the Eyes Test (RMET), the Toronto Alexithymia Scale (TAS-20), and the Profile of Mood States (POMS). An empathy-induction task involved videos of people experiencing violence. Saliva samples were collected at baseline, anticipatory, and post-task. Hormonal reactivity (AUCi) and total levels (AUCg) were calculated. Group comparisons used independent-samples t-tests, and associations between socio-affective measures and hormonal ratios were assessed via Pearson correlation. **Results**: IPVp scored lower on the RMET [t_(22)_ = –2.86, *p* = 0.009] and higher on the TAS-20 [t_(22)_ = 2.18, *p* = 0.040], with no group differences on the POMS. RMET scores correlated negatively with T/C AUCg [r_(22)_ = –0.46, *p* = 0.024] and positively with OXT/T AUCi [r_(22)_ = 0.56, *p* = 0.004]. TAS scores correlated negatively with OXT/T AUCg [r_(22)_ = –0.46, *p* = 0.023], and POMS scores correlated negatively with OXT/C AUCi [ρ_(24)_ = –0.42, *p* = 0.041]. **Conclusions**: Socio-affective impairments in IPVp appear to be linked to differential hormonal ratios, which may affect functions relevant to social cognition. This profile may hinder processing and responding to social cues during interpersonal conflict, sustaining violent behavior. Interventions enhancing sensitivity to safe social cues and socioemotional regulation may reduce recidivism in IPVp.

**P10. Cognitive Alteration and Gut Microbiome Shifts in Post-Covid-19 Condition**

Mar Ariza^1,2^, Marc Llirós Dupré^3,4^, Neus Cano^2,5^, Barbara Segura^2,6,7^, Javier Bejar^8^, Cristian Barrué^8^, C. Ulises Cortés^8^, C Junqué^5,6,7^, M Garolera^2,5,9^

^1^Unitat de Psicologia Mèdica, Departament de Medicina, Universitat de Barcelona (UB), 08036 Barcelona, Spain

^2^Grup de Recerca en Cervell, Cognició i Conducta, Consorci Sanitari de Terrassa (CST), 08221 Terrassa, Spain

^3^Bioinformatics and Bioimaging (BI-SQUARED) research group, Biosciences department, Faculty of Science, Technology and Engineering, Universitat de Vic – Universitat Central de Catalunya, 08500 Vic, Catalunya, Spain

^4^Bioinformatics and Bioimaging (BI-SQUARED) research group, Institut de Recerca i Innovació en Ciències de la Vida i de la Salut a la Catalunya Central (IRIS-CC), Universitat de Vic – Universitat Central de Catalunya, 08500 Vic, Catalunya, Spain

^5^Departament de Psicologia, Facultat de Medicina i Ciències de la Salut, Universitat Internacional de Catalunya (UIC), 08195 Sant Cugat del Vallès, Barcelona, Spain

^6^Institute of Neurosciences, Universitat de Barcelona, 08036 Barcelona, Spain

^7^Institut d’Investigacions Biomèdiques August Pi i Sunyer (IDIBAPS), 08036 Barcelona, Spain

^8^Department of Computer Science, Universitat Politècnica de Catalunya (UPC) - BarcelonaTech, 08034 Barcelona, Catalunya, Spain

^9^Neuropsychology Unit, Consorci Sanitari de Terrassa- Hospital Universitari (CST), 08221 Terrassa, Spain

**Introduction**: Post-Coronavirus Disease 2019 Condition (PCC) is characterized by persistent symptoms including fatigue, cognitive impairment, respiratory and cardiovascular problems, gastrointestinal disturbances, depression and anxiety, and alterations in smell and taste. Nearly 12.5% of those infected with SARS-CoV-2 will develop PCC, and many present cognitive dysfunctions linked to the infection. The aim of this study was to explore the relationship between cognitive dysfunction and gut microbiome composition in a subset of PCC subjects. **Methodology**: We included 159 PCC patients (mild and severe) and 33 healthy controls (HC) from the DIANA project (ClinicalTrials.gov NCT05307549). All participants underwent 16S rRNA gene sequencing (Ion-Torrent PGM platform) and a comprehensive neuropsychological assessment covering all major cognitive domains. From this battery, a global cognitive impairment index was calculated (scores ≥1.5 SD below normative values, ratio of impaired tests/total tests) and dichotomized into altered vs. non-altered cognition. Microbiome differences were first analysed across clinical groups (HC, mild, severe) and then by cognitive status using Kruskal–Wallis tests followed by pairwise Wilcoxon comparisons. **Results**: The 20 most abundant ASVs (≈40% of all recovered ASVs) were assigned to Bacteroidetes (19.0%), Prevotella_9 (4.6%), Faecalibacterium (2.8%), Dialister, Alistipes, Parabacteroides, Prevotellaceae, UCG-002, Agathobacter, and Escherichia-Shigellam genera. Significant differences between PCC subgroups and HC were found for three genera. Prevotella_9 differed between mild and severe patients (*p* = 0.003), Faecalibacterium between mild and HC (*p* = 0.014), and UCG-002 both between mild and HC (*p* = 0.0098) and between mild and severe (*p* = 0.032). No global differences in richness or diversity were observed between PCC and HC. However, stratification by cognitive status revealed compositional shifts, with significant differences in Bacteroides (*p* = 0.017), Prevotella_9 (*p* = 0.010), and Parabacteroides (*p* = 0.015). **Conclusions**: This study provides novel evidence linking cognitive impairment and gut microbiome alterations in PCC. These findings highlight the need for further research to clarify causal pathways and assess potential therapeutic implications.

**P11. Maternal Separation Induces Sex-Specific Changes in Brain Oxidative Metabolism, Monoamine Activity, and Cytokine Expression**

Carolina González-Mateos^1,2^, Andrea Fernández-Blanco^1,2^, Héctor González-Pardo^1,2,3^, Eneritz Gómez-Lázaro^4^, Nélida M. Conejo^1,2,3^

^1^Laboratory of Neuroscience, Department of Psychology, Faculty of Psychology, University of Oviedo, 33003 Oviedo, Spain

^2^Institute of Neuroscience of the Principality of Asturias (INEUROPA), University of Oviedo, 33006 Oviedo, Spain

^3^Institute of Health of the Principality of Asturias (ISPA), 33011 Oviedo, Spain

^4^Department of Basic Psychological Processes and their Development, Basque Country University, 20018 San Sebastian, Spain

**Introduction**: Early-life stress (ELS) encompasses experiences of neglect and maltreatment that critically shape brain development and increase vulnerability to mental and physical disorders in adulthood. Adverse early experiences have been associated with a higher risk of depression, anxiety, substance use, and cognitive impairment, as well as systemic conditions such as obesity, cardiovascular diseases, and neurodegeneration. Animal models allow for the controlled study of these mechanisms, and maternal separation (MS) is a well-established paradigm for simulating psychosocial ELS. **Methodology**: We investigated the long-term, sex-specific effects of prolonged MS (4h/day, postnatal days 1-21) on brain mitochondrial function, monoamine levels, and neuroinflammation in adult Wistar rats males and females. Mitochondrial oxidative metabolism was quantified using cytochrome c oxidase (CCO) histochemistry, while monoaminergic activity and cytokine expression were assessed by HPLC and RT-qPCR, respectively. **Results**: Our results revealed a marked reduction in CCO activity in the prefrontal cortex, hippocampus, and nucleus accumbens shell of MS females compared with controls, whereas males showed less pronounced metabolic changes. In addition, sex-dependent differences were observed across both rearing conditions, affecting not only CCO activity, but also brain monoamine levels and turnover across brain regions. These findings indicate that MS affected neurotransmitter turnover differently in males and females. Furthermore, neuroinflammatory responses were sexually dimorphic: MS males exhibited elevated IL-6 and TNF-α expression in the prefrontal cortex and hippocampus, whereas MS females showed increased Il-6 levels selectively in the striatum. **Conclusions**: These findings highlight the complex, region- and sex-dependent neurobiological consequences of ELS, underscoring the importance of including both sexes in preclinical models to improve translational relevance for psychiatric and neurodevelopmental research.

**P12. Preliminary Design of a Psychometric Scale for Stress in Oncology Patients: Comparison With GAD-7**.

M. J. Montes Lozano^1^, R. A. Montoyo Antón^1^, S. Ortiz Montes^2^, B. López Mármol^3^

^1^Hospital General Universitario de Alicante, 03010 Alicante, Spain

^2^University of Alicante, 03690 Alicante, Spain

^3^Hospital Universitario Santa Lucía, 30202 Cartagena, Spain

**Introduction**: Stress is highly prevalent among oncology patients and may evolve into generalized anxiety and depression if it is not identified early. However, nursing practice still lacks a brief, specific psychometric tool to screen stress in this population. We designed a pilot stress questionnaire and examined its reliability and its relationship with the validated GAD-7 as a reference anxiety measure. **Methodology**: Cross-sectional pilot study in n = 54 oncology patients. Instruments: (i) a pilot stress questionnaire hypothesized to include three domains—overload, somatic symptoms, and coping difficulties—and (ii) GAD-7 for generalized anxiety. Analyses (JASP) comprised internal consistency (Cronbach’s α, McDonald’s ω), exploratory factor analysis, linear regression of stress factors on GAD-7, and paired-samples t-tests. **Results**: Internal consistency was good for the overall pilot scale (α = 0.865, ω = 0.868). By factor: F1 Overload α = 0.865, F2 Somatic α = 0.772, F3 Coping α = 0.615. In linear regression, stress factors did not significantly predict GAD-7 (R^2^ = 0.039, *p* = 0.575). A paired *t*-test showed significant differences between F3 (Coping) and GAD-7 (t_(53)_ = –30.32, *p* < 0.001). Interpretation: the pilot stress questionnaire captures dimensions that are distinct from generalized anxiety, suggesting complementary information for oncology care. **Conclusions**: The pilot scale shows promising reliability and constructs signals different from GAD-7, supporting continued development and validation. A brief, nursing-oriented stress tool may aid early detection and timely psychosocial support in oncology settings. Next steps include confirmatory factor analysis, test–retest reliability, convergent/discriminant validity, and cut-off calibration against clinical endpoints.

**P13. Sex-Specific Microglial Responses to Juvenile and Adult Stress: Implications for Depression Vulnerability**

Patricia Chaves-Peña^1^, Jose Munoz-Martin^2,3^, María Inmaculada Infantes-López^2,3^, Víctor Martin-Aguiar^1^, Andrea Nieto-Quero^2,3^, Margarita Pérez-Martín^2,3^, Carmen Pedraza^1,3^

^1^Departamento de Psicobiología y Metodología de las Ciencias del Comportamiento, Universidad de Málaga, 29010 Málaga, España

^2^Departamento de Biología Celular, Genética y Fisiología, Universidad de Málaga, 29071 Málaga, España

^3^Instituto de Investigación Biomédica de Málaga y Plataforma en Nanomedicina-IBIMA, plataforma Bionand, 29010 Málaga, España

**Introduction**: Stress during sensitive developmental periods promotes long-lasting microglial sensitization, increasing vulnerability to subsequent stressors in adulthood and precipitating depressive-like symptoms. Additionally, it is known that depression is more common in females (5.1%) than in males (3.6%). Consequently, we investigated potential sexual differences in the effects of stress on microglial morphology and their associated inflammatory profile. This study aimed to explore sexual differences in microglial sensitization induced by juvenile stress and its consequences following adult stress exposure. **Methodology**: Male and female C57BL/6J mice were subjected to two stress protocols, the first in the juvenile period and the second in adulthood. Microglial morphology was assessed through morphometric analyses, and pro- and anti-inflammatory cytokine expression profiles were quantified. **Results**: Our findings indicate that juvenile stress sensitizes microglia in both sexes, with adult stress later unmasking sex-specific phenotypes. In males, juvenile and adult stress independently reduced the aspect ratio, indicative of a rounder soma and a more activated microglial phenotype. In females, adult stress increased soma area and disrupted cellular regularity. Similarly, a differential expression of the cytokines profile was observed between sexes. In males, adult stress elicited pro-inflammatory response with increased IFN-γ expression, whereas in females, cumulative stress promoted upregulation of VEGFa, consistent with remodeling and growth factor signaling rather than classical inflammation. In summary, juvenile stress establishes a state of microglial sensitization that predisposes to sex-specific responses when a second challenge occurs in adulthood. Males preferentially exhibit pro-inflammatory activation, while females show changes related to cellular remodeling. These dimorphic microglial trajectories may underlie the differential vulnerability to depression observed between sexes. **Conclusions**: Microglial sensitization during early life emerges as a critical mechanism shaping long-term neuroimmune responses to stress, providing insight into the sex-specific basis of mood disorders.

**P14. Physical Exercise and Perinatal Stress: Psychological vs. Biological Outcomes**

Miguel Ángel Baos-González^1,2^, Javier De Echarri-Lorente^1,2^, Rocío RodríguezValdés^1,2^, Borja Romero-González^3^, Raquel González-Pérez^4^, María Isabel Peralta-Ramírez^1,2^

^1^Mind, Brain and Behavior Research Center (CIMCYC), University of Granada, 18071 Granada, Spain

^2^Department of Personality, Assessment, and Psychological Treatments, Faculty of Psychology, University of Granada, 18071 Granada, Spain

^3^Faculty of Education of Soria, University of Valladolid, 42004 Soria, Spain

^4^Department of Pharmacology, Centro de Investigación Biomédica en Red de Enfermedades Hepáticas y Digestivas (CIBERehd), School of Pharmacy, Instituto de Investigación Biosanitaria ibs. Granada, University of Granada, 18012 Granada, Spain

**Introduction**: Pregnant women often experience elevated anxiety and stress, reflected in hair cortisol concentrations (HCC). These symptoms impair quality of life and are linked to postpartum depression, pregnancy complications, and offspring development. Physical exercise has been shown to reduce anxiety during pregnancy, but its effects on stress are less clear. Research on perceived stress has largely focused on yoga interventions, while findings on HCC, a biomarker of chronic stress, remain inconsistent. Some studies report no associations, others reductions in HCC with frequent exercise, and others increases with high-intensity training. This study examined associations between physical activity, anxiety, perceived stress, and HCC during pregnancy. **Methodology**: A total of 574 pregnant women (M = 33.22 years, SD = 5.16) participated. They reported exercise engagement and weekly hours. Stress was assessed with the Perceived Stress Scale, anxiety with the SCL-90 subscale, and HCC from 3-cm hair strands. Assessments were conducted across all trimesters, using mean values. Spearman correlations and independent samples t-tests were applied, with log-transformed HCC. **Results**: Women engaging in physical exercise reported lower perceived stress (t = 4.119, *p* < 0.001, M = 24.33 vs. 26.18) and lower anxiety (t = 2.326, *p* = 0.020, M = 61.01 vs. 65.73 percentiles) compared to non-exercisers. No differences were observed in HCC (t = –0.143, *p* = 0.886, M = 5.18 vs. 5.17). Greater weekly exercise hours were correlated with lower perceived stress (r = –0.164, *p* < 0.001) and anxiety (r = –0.097, *p* = 0.025), but not with HCC (r = –0.030, *p* = 0.469). **Conclusions**: Physical exercise during pregnancy is associated with lower perceived stress and anxiety, suggesting a protective role for maternal physical and mental health. No associations with HCC were found, indicating that inconsistencies in prior research may reflect differences in exercise type, intensity, or methodology.

**P15. Pre-pregnancy BMI as a Risk Factor: Associations With Maternal Hair Cortisol and Child Psychopathology at Age Two**

Javier De Echarri Lorente^1,2^, Miguel Ángel Baos González^1,2^, Rocío Valdés Rodríguez^1,2^, Carolina Mariño-Narváez^3^, Raquel González Pérez^4^, María Isabel Peralta Ramírez^1,2^

^1^Department of Personality, Assessment and Psychological Treatment, University of Granada, 18071 Granada, Spain

^2^Mind, Brain and Behaviour Research Centre, University of Granada, 18071 Granada, Spain

^3^Faculty of Education Sciences and Humanities, International University of La Rioja, 26006 Logroño, Spain

^4^Department of Pharmacology, Centro de Investigación Biomédica en Red de Enfermedades Hepáticas y Digestivas (CIBERehd), School of Pharmacy, Instituto de Investigación Biosanitaria ibs. Granada, University of Granada, 18012 Granada, Spain

**Introduction**: Pre-pregnancy maternal body mass index (BMI) has been established as a key variable in pregnancy outcomes. Elevated BMI increases the risk of adverse maternal health conditions, including gestational diabetes, miscarriage, hypertension, and a higher likelihood of cesarean delivery. These risks also extend to newborns, who face greater complications during delivery and an increased risk of macrosomia. The present study aimed to examine the interaction between maternal BMI and biomarkers such as hair cortisol during pregnancy, as well as psychological variables including depression and perceived stress, and their association with child psychopathology at age two. **Methodology**: The sample comprised 100 pregnant women from the Gestastress-Childstress cohort, with longitudinal follow-up of their children at two years of age. Maternal body weight adequacy was evaluated by calculating pre-pregnancy BMI, maternal psychopathology during pregnancy was assessed using the Symptom Checklist-90-R (SCL-90-R), and chronic stress was measured throught hair cortisol concentrations. Child psychopathology was evaluated using the Child Behavior Checklist (CBCL). **Results**: Results indicated that higher pre-pregnancy BMI was associated with elevated hair cortisol concentrations (r = 0.089, *p* = 0.019), as well as higher internalizing (r = 0.217, *p* = 0.032) and externalizing problems (r = 0.279, *p* = 0.005) in children at two years of age. **Conclusions**: These findings are consistent with previous research, highlighting elevated pre-pregnancy BMI as a risk factor not only for heightened physiological stress responses during pregnancy but also as a predisposing condition that, in interaction with other variables, contributes to the emergence of early psychopathological symptoms in children.

**P16. Progressive Inhibition of Adult Hippocampal Neurogenesis by Temozolomide and Its Consolidation After a Rest Period**.

Alejandro Zea-Doña^1^, Patricia Chaves-Peña^1^, Víctor Martín-Aguiar^2^, Estela del Mar Sosa-Osorio^1^, Margarita Pérez-Martín^1,3^, Carmen Pedraza^2,3^

^1^Department of Cell Biology, Genetics and Physiology, University of Malaga, 29071 Malaga, Andalucia, Spain

^2^Department of Psychobiology and Methodologies of Behavioral Sciences, University of Malaga, 29071 Malaga, Andalucia, Spain

^3^Biomedical Research Institute of Malaga (IBIMA) Plataforma BIONAND, 29010 Malaga, Spain

**Introduction**: Adult hippocampal neurogenesis is increasingly recognized as a critical modulator of brain plasticity, emotional regulation, and neuroimmune interactions. Disruption of this process has been associated with stress-related psychopathology, in which microglial activation and inflammatory imbalance play central roles. Pharmacological inhibition of neurogenesis with agents such as temozolomide (TMZ) offers a valuable model to investigate how reduced neuronal turnover influences microglial reactivity and the neuroinflammatory milieu. However, the temporal dynamics and persistence of TMZ-induced inhibition remain poorly defined. In this study, we evaluated the effects of TMZ on hippocampal cell proliferation and survival using sequential administration of thymidine analogs (CldU, IdU) and the proliferation marker Ki-67. **Methodology**: Mice were assigned to three experimental groups: vehicle, TMZ, and TMZ followed by a 10-day drug-free recovery period. TMZ was administered intraperitoneally over three weeks in cycles of 3 consecutive days of injections followed by 4 days of rest per week. CldU was injected on day 4 of the second week, and IdU on day 4 of the third week. Animals from the vehicle and TMZ groups were perfused at the beginning of week 4, while the TMZ + rest group was perfused at the start of week 5. **Results**: Results indicate that TMZ does not significantly reduce Ki-67 expression immediately after the three-week treatment. However, a marked decrease in cell proliferation emerges after the recovery period, as evidenced by reduced Ki-67 and IdU labeling. Survival analysis also suggests groupdependent differences, pending further validation. These findings suggest that TMZ-induced inhibition of neurogenesis develops progressively and becomes consolidated after treatment cessation. This model provides a robust framework to study how impaired neurogenesis shapes microglial phenotypes and inflammatory signaling. Ongoing analyses include microglial characterization via Iba-1 immunohistochemistry (density and morphology), and quantification of cytokines (IL-1β, IL-6, TNF-α, IL-10, IGF-1, BDNF) using RT-qPCR and Luminex assays. **Conclusions**: Integration of these molecular findings with behavioral data will help elucidate the role of neurogenesis–microglia interactions in stress-related vulnerability.

**P17. Analysis of the Association Between Natural Killer And Immunoglobulin A Levels and Anger: Conclusions of a Meta-Analytic Approach**

Ángel Romero Martínez^1^, Carolina Sarrate-Costa^1^, Luis Moya-Albiol^1^

^1^Department of Psychobiology, Faculty of Psychology, University of Valencia, 46010 Valencia, Spain

**Introduction**: The relationship between various immune system indicators, such as lymphocytes and antibodies, and psychological states such as anger has sparked significant debate in the scientific community. Conflicting evidence exists regarding the direction of the association between specific immune parameters and state anger. However, no meta-analysis has yet examined all available scientific literature to determine the direction of the relationship between natural killer (NK) and immunoglobulin A (IgA) levels and anger in humans. **Methodology**: This study conducted a meta-analysis in accordance with the Preferred Reporting Items for Systematic Reviews and Meta-Analyses (PRISMA) guidelines. After initially identifying 315 sources through three databases—PubMed, Scopus, and Web of Knowledge—we ultimately included 12 publications. **Results**: Based on the studies included, NK levels were not significantly associated with anger in men and women (the correlation coefficient was 0.08, ranging from –0.59 to 0.68 in approximately 202 participants), and IgA levels in maternal milk were also not significantly associated with anger in women (the correlation coefficient was 0.07, ranging from –0.33 to 0.46 in approximately 280 participants). However, salivary IgA levels were significantly and positively related to anger levels in both men and women (the correlation coefficient was 0.14, ranging from 0.06 to 0.23 in approximately 282 participants). **Conclusions**: We found some support for a positive association between salivary IgA levels and anger. Future studies should address the limitations of current research to clarify how anger may influence immune functioning.

**P18. Extraversion Reduces Cortisol in Patients With Fibromyalgia by Decreasing Perceived Impact of the Disease**

Dolores Santiago^1^, Ana M. Contreras-Merino^1^, Pablo de la Coba^1^, Carmen Gálvez-Sánchez^1^, Casandra I. Montoro^1^, Gustavo A. Reyes del Paso^1^

^1^University of Jaén, 23071 Jaén, Spain

**Introduction**: Fibromyalgia (FM) is a chronic pain condition characterized by widespread musculoskeletal pain, fatigue, and insomnia, often accompanied by anxiety and depression. Personality traits have been proposed as potential aggravators or attenuators of clinical symptoms in FM. Specifically, extraversion has been consistently associated with better psychological outcomes, including lower perceived stress, reduced anxiety and depression, enhanced coping strategies, and improved quality of life. FM has been linked to hypothalamic-pituitary-adrenal axis dysregulation. Some studies report lower baseline cortisol levels in FM patients, particularly in urine, saliva, and morning awakening responses, as well as a blunted cortisol reaction to acute stressors. These findings suggest that both psychological and physiological mechanisms may interact to influence the overall impact of the disease. The aim of this study was to examine the influence of extraversion on cortisol levels in patients with FM and to explore how this relationship contributes to the perceived impact of the disease. **Methodology**: Hair cortisol concentrations, fibromyalgia impact, and extraversion were assessed in 48 patients with FM and 31 healthy controls. **Results**: Cortisol levels were positively associated with scores in the Fibromyalgia Impact Questionnaire (FIQ), indicating that greater disease impact corresponded to higher cortisol concentrations. Extraversion was negatively associated with both FIQ scores and cortisol levels. Mediation analyses further revealed that FIQ scores acted as a moderating variable in the relationship between extraversion and cortisol, suggesting that extroversion reduced the impact of FM and the lower disease burden decreses cortisol secretion. **Conclusions**: Extraversion appears to act as a protective factor in FM. This finding highlights the potential of interventions targeting personality-related coping strategies, such as promoting social engagement, fostering positive affect, and developing adaptive stress management skills, to reduce psychological stress, modulate cortisol levels, and ultimately alleviate the perceived impact and burden of the disease. Enhancing these protective traits may represent a promising complementary approach in FM management.

**P19. Bariatrics Surgery and the Mind-Body Connection: A Protocol for the Longitudinal Assessment of the Impact of Bariatric Surgery on Psychological Wellbeing**

Lorena Andreu-Lucena^1^, Marco-Alacid, Cristian^2^, Abad-González, Ángel Luis^3,4^, Pérez-Climent, Nieves^2^, Amrani, Rahma^3,4^, Castillo-García, María Trinidad^3,4^, Sánchez-Pacheco Tardón, Myriam^3,4^, Santos-Ruiz, Ana^1,4^

^1^University of Alicante, 03690 San Vicente del Raspeig, Alicante, Spain

^2^Virgen de los Lirios Hospital, 03804 Alcoy, Alicante, Spain

^3^Dr. Balmis General University Hospital, 03010 Alicante, Spain

^4^Alicante Institute for Health and Biomedical Research (ISABIAL), 03010 Alicante, Spain

**Introduction**: Obesity is a growing public health concern worldwide, affecting 58% of the adult European population. In cases of morbid obesity, bariatric surgery is considered an effective intervention to achieve significant and sustained weight loss, as well as improvements in quality of life and psychological well-being. However, these changes have not been extensively investigated in the long term, as psychological well-being has been shown to decline after two years. This research protocol aims to analyse the effects of bariatric surgery on the mental health of patients with morbid obesity, as well as on hair cortisol concentrations, through a three-year longitudinal follow-up. One year after bariatric surgery, patients will experience a significant improvement in the psychological variables under study. Moreover, low pre-surgery hair cortisol concentrations will predict successful postoperative weight loss. However, deterioration in psychological variables is expected at two- and three-year follow-up, accompanied by partial weight regain. **Methodology**: Observational, prospective study with a threeyear follow-up of morbidly obese patients undergoing bariatric surgery. The study will be conducted in two hospitals in Alicante, and patients scheduled to undergo either gastric bypass or sleeve gastrectomy will be selected. Eligible participants will be adults with a body mass index (BMI) ≥40 who have not previously undergone bariatric surgery. Symptoms of anxiety, depression, stress, binge eating disorder, impulsivity, emotional eating, dietary behaviour, and hair cortisol concentrations will be assessed at five time points: three months pre-surgery (T0), one-month post-surgery (T1), and at one-year (T2), two-years (T3), and three-years (T4) post-surgery. The results may reveal the short-term psychological benefits of bariatric surgery, along with the expected medium- to long-term deterioration. In addition, the psychological variables mediating bariatric surgery outcomes during follow-up would be identified. This would allow for the detection of dimensions requiring additional attention in the preoperative and postoperative management of these patients.

**P20. Adolescent Stress Induces A Depressive-Like Phenotype Accompanied By Metabolic And Neuroimmune Disruptions**

Irene Ferreres Álvarez^1^, Sílvia Castany-Quintana^1^, Elida Alechaga Silva^2^, Óscar Pozo Mendoza^2^, Arnau Busquets Garcia^1^

^1^Hospital del Mar Research Institute, Cell-type mechanisms in normal and pathological behaviour, PRBB Building, 08003 Barcelona, Spain

^2^Hospital del Mar Research Institute, Applied metabolomics, PRBB Building, 08003 Barcelona, Spain

**Introduction**: The understanding of stress-related psychiatric disorders is needed to explain the alarming high rise of these disorders in adolescence. Indeed, adolescent individuals are more sensitive to certain stressors, emphasizing the importance of adolescence as a vulnerable period for the onset of stress-related psychiatric disorders. Chronic stress has been linked with neuroinflammation, which is thought to be partly mediated by specific alterations in glial cells. Nevertheless, the mechanisms behind this disruption remain unclear, since glial cells have long been disregarded. In this sense, we hypothesized that disruptions in the kynurenine pathway in glial cells, which metabolizes the essential amino acid tryptophan and is upregulated by inflammatory signals, could explain the impact of adolescent chronic stress on behaviour. **Methodology**: To mimic both the psychological and biological components of stress during adolescence, we used a double hit model consisting of a chronic stress protocol (Postnatal day (PD)30 - PD58) in male and female mice, which combines social isolation with the oral administration of corticosterone. Following this protocol, a subset of behavioural tasks were performed to assess anxiety-like behaviour (Elevated Plus Maze), sociability (V-SOC task and direct social interaction in an Open field) and depressive-like behaviour (Sucrose Splash Test and Forced Swimming Test). **Results**: Chronic stressed mice presented behavioural alterations such as depressive-like behaviour and anhedonia. Moreover, after the behavioural evaluation, we extracted different brain regions to analyse potential changes related to neuroinflammation and the kynurenine pathway. **Conclusions**: Altogether, this study provides novel behavioural and neuroimmune insights linking adolescent stress with specific behavioural alterations. It remains to be investigated the downstream effect of this metabolic disruption and the potential use of therapies that tackle this pathway.

**P21. Allostatic Load and Socioeconomic Inequalities in Depressive Symptoms Among Cancer Survivors**

Alicia Rubio Pilares^1,2^, Dafina Petrova^2,3,4^, Blanca Madrid Pérez-Esparza^1,2^, María-José Sánchez^2,3,4^, Rocío García-Retamero^1^

^1^Universidad de Granada, 18071 Granada,Spain

^2^Instituto de Investigación Biosanitaria ibs. GRANADA, 18012 Granada, Spain

^3^Escuela Andaluza de Salud Pública, 18011 Granada, Spain

^4^CIBER de Epidemiología y Salud Pública (CIBERESP), 28029 Madrid, Spain

**Introduction**: Cancer survivors, particularly those with lower socioeconomic status (SES), are at increased risk of depression. Chronic stress may contribute to this vulnerability through cumulative physiological dysregulation, measurable with the allostatic load index. We investigated whether allostatic load mediates or moderates the association between SES and depressive symptoms among cancer survivors. **Methodology**: We analyzed data from 691 adults with history of cancer (excluding non-melanoma skin cancer) who participated in the National Health and Nutrition Examination Survey 2017–2020. Allostatic load was derived from 10 biomarkers (blood pressure, pulse, glycohemoglobin, creatinine clearance, HDL, total cholesterol, albumin, white blood cell count, and C-reactive protein) combined with medication use. SES was assessed using education and income-to-poverty ratio. Depressive symptoms were measured with the Patient Health Questionnaire-9. Multiple regression models, adjusted for sex, age, marital status, and cancer type, tested whether allostatic load mediated or moderated SES–depression associations. **Results**: Higher allostatic load was significantly associated with more depressive symptoms (B = 0.19, *p* < 0.001) and with lower income (B = 0.43, *p* < 0.001) and lower education (B = 0.43, *p* < 0.001). Survivors with lower income reported more depressive symptoms, and this association was partially mediated by higher allostatic load (mediated effect = 0.07, 95% CI 0.03–0.10). No mediation was observed for education. The effect of allostatic load on depressive symptoms was consistent across income groups, indicating no moderation. **Conclusions**: Among cancer survivors, allostatic load may be a pathway linking socio-economic disadvantage to depressive symptoms. These findings highlight the potential relevance of biological stress markers for survivorship care and addressing socioeconomic inequalities in mental health in this vulnerable population.

**P22. Sex-Specific Microglial and Microbiota Responses to Chronic Social Stress**

E.M. Sosa Osorio^1^, I Infantes López^1^, E Zambrana-Infantes^1^, J Muñoz-Martín^1,2^, P Chaves-Peña^1^, A Nieto-Quero^1^, M Domínguez-Maqueda^1^, I Cerezo-Ortega^1^, M.A. Moriñigo^1^, M Pérez-Martín^1,2^, C Pedraza-Benítez^1,2^

^1^Departamento de Psicobiología y Ciencias del Comportamiento, Universidad de Málaga, 29071 Málaga, Spain

^2^Instituto de Investigación Biomédica de Málaga y Plataforma en Nanomedicina (IBIMA Plataforma BIONAND), 29010 Málaga, Spain

**Introduction**: Mood disorders pose a serious health threat in society, affecting 1 in 8 people globally. While chronic stress is considered a major risk factor in the development of mental illnesses, the neurobiological mechanisms underlying sex-specific vulnerability are not yet fully understood. Here, we examined the impact of Social Defeat Stress (SDS) on microglial morphology and gut microbiota in male and female mice. **Methodology**: Microglial soma and branching were quantified by morphometry, and clustering analyses were applied to ramification patterns. The microbial composition was evaluated using α- and β-diversity, as well as relative abundance. Mediation models explored potential links among SDS, microbiota, and microglia. **Results**: In males, SDS induced hyper-ramified, denser microglia and significant shifts in microbial β- diversity and composition across taxonomic levels. In females, microglia acquired an amoeboid profile, but microbiota remained stable across groups. Mediation analyses suggested that in males, SDS-driven microbial changes were associated with microglial remodelling, whereas in females’ microglial alterations occurred more directly, with weaker microbiota involvement. **Conclusions**: These findings highlight the importance of sex in shaping neuroimmune and microbial responses to stress. Such insights may help explain sex differences in stress-related disorders and guide interventions targeting microbiota-brain interactions.

**P23. Behavioral Weight Loss Programs Reduce Stress and Improve Quality of Life**

Francisco Javier Pérez-Comino^1^, Lucía Solier-López^1^, Andrea Bernat-Villena^1^, Raquel González-González^1^, Luz Stella Algarra-López^1^, Alfonso Caracuel^1^, Raquel Vilar-López^1^

^1^Mind, Brain and Behavior Research Center (CIMCYC), University of Granada, 18071 Granada, Spain

**Introduction**: Excess weight (EW) has tripled in recent years. An elevated Body Mass Index (BMI) is a risk factor for multimorbidity, and chronic diseases. Moreover, EW is associated with higher stress and overall reduced quality of life. Our previous work has shown a 6-week behavioral weight loss program based on Motivational Interview, individualized diet and physical exercise to be effective in improving anthropometric measures (such as BMI and waist-to-height ratio). Based on these findings, the aim of the present study was to determine whether the program was associated with improvements in stress and quality of life, and to explore the association between stress and quality of life in people with EW. **Methodology**: The sample included 148 individuals with EW (85.1% women), mean age 44 years (SD = 6.78), mean education 15 years (SD = 5.34), and mean BMI 31.61 (SD = 4.01). Stress and quality of life were assessed at baseline, post-intervention, and at 3- and 6-month follow-ups using the Perceived Stress Scale (PSS) and the SF-36 Health Survey. **Results**: Repeated measures ANOVA revealed a significant effect of time on stress (F = 5.08, *p* = 0.007) and quality of life (F = 6.79, *p* = 0.010). A linear regression showed that stress reduction significantly predicted an increase in overall quality of life (B = –1.54, SE = 0.24, t = –6.54, *p* = 0.001), physical (F = 4.09, *p* = 0.046, R^2^ = 0.036), and mental health-related quality of life (F = 75.54, *p* = 0.001, R^2^ = 0.407). **Conclusions**: Participation in the weight loss program led to reductions in stress and improvements in quality of life (both physical and especially mental-health related) in individuals with EW. Furthermore, stress changes predicted quality of life improvements.

**P24. Impact of Stress During the Juvenile Period in the Development of Adult Depression and Anxiety: Neuroinflammatory Findings**

Víctor Martín-Aguiar^1^, Alejandro Zea-Dona^1^, Jose Munoz-Martin^1,2^, Patricia Chaves-Peña^1^, Margarita Pérez-Martín^1,2^, Carmen Pedraza^1,2^

^1^University of Malaga, 29071 Malaga, Andalucia, Spain

^2^Biomedical Research Institute of Malaga (IBIMA) Plataforma BIONAND, 29010 Malaga, Spain

**Introduction**: Juvenile stress represents a critical risk factor for adult depression, as it disrupts brain maturation and hypothalamic-pituitary-adrenal (HPA) axis regulation. Early adversity increases vulnerability to depressive symptoms, worsens treatment outcomes, and contributes to clinical heterogeneity. Identifying neuroimmune-related biomarkers that predict vulnerability or resistance is therefore a major research priority. Candidate biomarkers include inflammatory alterations, HPA axis dysregulation, and epigenetic modifications. Their expression is further modulated by sex, type, and duration of stress, highlighting the importance of tailored and preventive interventions. **Methodology**: We have conducted a systematic review on the topic, selecting peer-review articles on animal models that included juvenile stress, a depression test in adulthood, and biomarkers related to the process that were written in English and Spanish. This review followed PRISMA and Cochrane guidelines and was registered in PROSPERO. **Results**: Of the 52 articles included, and among all biomarkers found, this contribution will focus on those 8 articles including related to neuroinflammation and the immune system. Chronic and acute stress paradigms consistently induced anxiety and depressive-like behaviors, with sex-specific different patterns, with males often showing more anxiety and females displaying altered exploratory and risk-taking behaviors. Neuroinflammatory changes were robust across studies, with increased microglial activation and pro-inflammatory cytokines (IL-1B, IL-6, TNF-a) in hippocampus, PFC, and NAc, with one study even reporting an affected microbiota with an inflammatory profile. Pharmacological interventions such as propranolol or minocycline effectively reduced anxiety/depressive phenotypes and suppressed neuroinflammation. **Conclusions**: The objective of this review is to contribute to developing evidence-based and potentially individualized approaches to prevention and intervention in those at higher risk.

**P25. Neuroimmune Profile and Inflammatory Biomarkers in Fibromyalgia Syndrome: A Review of Recent Evidence**

Belén Moreno^1^, Gustavo A. Reyes del Paso^1^, Carmen María Galvez-Sánchez^2^

^1^University of Jaén, 23071 Jaén, Spain

^2^University of Murcia, Murcia, Spain

**Introduction**: Fibromyalgia Syndrome (FMS) is a chronic disorder characterized by widespread musculoskeletal pain, persistent fatigue, and sleep disturbances, frequently cooccurring with symptoms of anxiety and depression. Emerging evidence suggests that FMS involves complex neuroimmune interactions, with inflammation—particularly cytokines and chemokines—playing a key role. Neuroinflammation contributes to central sensitization and thus chronic pain, potentially driven by glial–immune cell interactions and modulated by gut microbiota. Additionally, dysregulation of the hypothalamic-pituitary-adrenal (HPA) axis and stress response pathways has been linked to altered immune activity and pain hypersensitivity. This review summarizes recent advances in the immunopathogenic understanding of FMS, focusing on innate and adaptive immune involvement. **Methodology**: A narrative review was conducted to provide an integrative overview of the topic. The SALSA framework (Search, Appraisal, Synthesis, and Analysis) guided the systematic search process. Inclusion criteria focused on English-language studies from PubMed, Scopus, and Web of Science involving adult populations diagnosed with FMS based on American College of Rheumatology (ACR) criteria. Reference lists were also screened to identify relevant literature. Keywords included “fibromyalgia syndrome”, “immune system”, “neuroinflammation”, “proinflammatory cytokines”, and “leukocytes”. A total of 37 articles were selected after quality assessment. **Results**: Studies reveal immune dysregulation in FMS, with elevated proinflammatory cytokines—mainly IL-6, IL-8, and TNF-α— that are associated with pain, fatigue, sleep disturbances, and depression. These cytokines drive neuroinflammation and central sensitization and are released by immune cells such as neutrophils and monocytes. IL-8 is a promising biomarker, uniquely elevated in FMS compared to other rheumatic diseases. While proinflammatory cytokines exacerbate symptoms, anti-inflammatory cytokines like IL-10 may help counterbalance inflammation. **Conclusions**: Research shows neuroimmune alterations in fibromyalgia, with specific cytokine patterns specific from other diseases. Inflammatory markers like cytokines correlate with symptom severity. While FM’s autoimmune status is unclear, a neuroimmune basis is likely. Further studies are needed to improve diagnosis and treatment.

**P26. Fatigue in Multiple Sclerosis: Psychosocial Variables and Diurnal Cortisol Secretion**

Daniel Gonzalez-Templado^1^, Leire Romarate^1^, Idoia Mendiburu^2,3^, Naiara Andrés^2,3^, Maialen Arruti^2,3^, Hirune Crespillo^2^, Ander Iriarte-Sarria^4,5^, Oscar Vegas^1^, Nora del Puerto^1^, Maider Muñoz-Culla^1,2^

^1^Department of Basic Psychological Processes and Their Development, University of the Basque Country (UPV/EHU), 20018 Donostia-San Sebastián, Spain

^2^Neuroimmunology Group, Neuroscience Area, Biogipuzkoa Health Research Institute, 20014 Donostia-San Sebastián, Spain

^3^Donostia University Hospital, 20014 Donostia-San Sebastián, Spain

^4^Department of Neurosciences, University of the Basque Country (UPV/EHU), 48940 Leioa, Spain

^5^Achucarro Basque Center for Neuroscience, 48940 Leioa, Spain

**Introduction**: Fatigue is one of the most prevalent and disabling symptoms in Multiple Sclerosis (MS), yet its pathophysiology remains unclear. Both psychosocial factors (distress, anxiety, depression) and hypothalamic-pituitary-adrenal (HPA) axis activity have been proposed as contributors. This pilot study examined the relationship between psychosocial variables, diurnal cortisol secretion, sex, and disability in patients with relapsing-remitting MS (RRMS). **Methodology**: A cross-sectional study was conducted with 52 RRMS patients (39 women, 13 men). Participants completed questionnaires assessing fatigue (MFIS), distress (IES-R), anxiety and depression (HADS), and disability (EDSS). Salivary cortisol was collected at four time points across one day to evaluate diurnal secretion and cortisol awakening response (CAR). Mann–Whitney U test and repeated measures ANOVA were conducted in the full sample and stratified by sex. **Results**: Patients with fatigue (n = 26) reported higher distress (*p* < 0.001), anxiety (*p* = 0.006), depression (*p* < 0.001), and disability (*p* = 0.049). No differences in cortisol secretion were observed between fatigue groups. However, two distinct CAR patterns emerged: positive (n = 37) and negative (n = 15). Participants with a negative CAR exhibited higher distress (*p* = 0.043), particularly on the intrusion-hyperactivity subscale (*p* = 0.023). Stratified analyses revealed that women with a negative CAR (n = 10) also reported higher anxiety and depression scores (*p* < 0.05) than women with a positive CAR (n = 29). A statistical trend was observed in these women, who reported higher total fatigue, and higher scores on the cognitive and psychosocial fatigue subscales (*p* < 0.1). **Conclusions**: Fatigue in MS is associated with psychosocial distress, anxiety and depressive symptomatology, and disability, while diurnal cortisol secretion shows no direct differences between fatigue groups. Altered CAR patterns, especially in women, appear to be linked to higher distress, anxiety, and depression, suggesting sex-specific pathways in HPA axis dysregulation. These findings underscore the importance of integrating psychosocial and biological perspectives in understanding MS-related fatigue and highlight the need for larger confirmatory studies, which are currently underway.

**P27. Impact of Breastfeeding on Infant Neurodevelopment: Cognition, Language, and Motor Skills at 6 Months**

Rocío Rodríguez Valdés^1^, Miguel Ángel Baos González^1^, Javier de Echarri Lorente^1^, Carolina Mariño Narváez^2^, Borja Romero González^4^, María Isabel Peralta Ramírez^1,2^

^1^Mind, Brain and Behavior Research Center (CIMCYC), University of Granada, 18071 Granada, Spain

^2^Department of Personality, Assessment and Psychological Treatment, Faculty of Psychology, University of Granada, 18071 Granada, Spain

^3^Department of Psychology, Faculty of Education and Humanities, International University of La Rioja, 26006 Logroño, Spain

^4^Department of Psychology, Faculty of Education, University of Valladolid, 42004 Soria, Spain

**Introduction**: Infant neurodevelopment is influenced by multiple biological and environmental factors, among which breastfeeding plays a central role. Several studies have shown that exclusive breastfeeding and its duration are beneficial for neural development, improving brain architecture, white matter maturation, and cognitive performance. The aim of this study was to analyze the relationship between type of breastfeeding and cognitive, language, and motor development in infants at six months of age, assessed with the Bayley battery. **Methodology**: The sample consisted of 132 infants aged six months. Participants were divided into two groups: exclusive breastfeeding and mixed/artificial feeding. Three periods of breastfeeding exposure were considered: 0–6 weeks (n = 101 exclusive, n = 31 mixed/artificial), 6 weeks–3 months (n = 97 exclusive, n = 35 mixed/artificial), and 3–6 months (n = 88 exclusive, n = 44 mixed/artificial). Neurodevelopment was assessed with the Bayley battery, which provides scaled scores in cognition, receptive and expressive communication, fine motor skills, and gross motor skills. *t*-tests were conducted, with homogeneity of variances tested using Levene’s test. **Results**: Significant differences were consistently found in the cognitive domain across all three periods, with the exclusively breastfed infants presenting higher scores (0–6 weeks: F = 7.23, *p* = 0.008, 6 weeks–3 months: F = 8.74, *p* = 0.004, 3–6 months: F = 9.72, *p* = 0.002). Regarding language, significant differences were observed in expressive communication from six weeks onwards (6 weeks–3 months: F = 5.02, *p* = 0.027, 3–6 months: F = 4.26, *p* = 0.041), while receptive communication differences appeared only in the 3–6-month period (F = 7.55, *p* = 0.007). In contrast, fine and gross motor skills did not show significant differences in any of the periods evaluated. **Conclusions**: Exclusive breastfeeding is associated with better cognitive performance from the earliest weeks of life, as well as with advantages in language development from six weeks up to six months. These findings suggest that breastfeeding, beyond its nutritional contribution, constitutes a protective factor that enhances early brain development, with relevant implications for infant health promotion and neurodevelopmental prevention strategies.

**P28. The Role of the Immune System in Human Aggressive Behavior: A Systematic Review**

Nora del Puerto-Golzarri^1^, Maider Muñoz-Culla^1^, Alina Díez-Solinska^2^

^1^Department of Basic Psychological Processes and their Development, University of the Basque Country (EHU), 20018 San Sebastian, Spain

^2^Health Sciences, Public University of Navarre (UPNA), 31006 Pamplona, Spain

**Introduction**: Aggressive behavior is a multifaceted phenomenon influenced by biological, psychological, and social factors. Increasing evidence highlights the immune system as a potential modulator of aggression, in line with psychoneuroimmunology research demonstrating bidirectional communication between the nervous and immune systems. This systematic review aimed to synthesize the available evidence on the relationship between immune markers and aggressive behavior in humans. **Methodology**: Following PRISMA guidelines, a systematic search was conducted in PubMed, PsycInfo, and Web of Science in February 2025. A total of 656 records were identified. After applying inclusion and exclusion criteria, 25 studies were included. **Results**: The studies reviewed varied widely in design, sample type (clinical and non-clinical populations, males and females, children to older adults), assessment tools for aggression, and immune markers evaluated (pro- and anti-inflammatory cytokines, immune cells, immunoglobulins, and enzymes). A considerable proportion of studies reported positive associations between aggression and pro-inflammatory markers such as IL-6, TNF-α, CRP, IFN-γ, and their ratios, as well as increased numbers of lymphocytes or higher immunoglobulin levels. Conversely, some studies identified negative associations (e.g., lower IL-6, IL-8, IL-1α in individuals with aggressive trajectories) or no significant relationship. Findings also suggested potential differences between clinical and non-clinical populations, and between normative versus pathological aggression. **Conclusions**: Evidence points toward a potential link between immune activation and aggressive behavior, particularly through pro-inflammatory pathways. However, inconsistent results underscore the complexity of this relationship, likely influenced by methodological heterogeneity, psychiatric comorbidity, and neuroendocrine factors such as cortisol and testosterone. Further longitudinal and mechanistic research is warranted to clarify causality and to explore the potential of immune markers as predictors or intervention targets in aggressive behavior.

**P29. Asssociation Between the BDNF*Val66Met* Polymorphism, Daily Cortisol Release and Personality Traits in Older Adults**

Leyre Castillejo Sanz^1^, Juan Ignacio Grec^1^, María Román Moreno^1^, Raúl Ballesta Barrera^1^, Sara García Herránz^1^, Cynthia Díaz-Silveira^2^, María del Carmen Díaz Mardomingo^1^, Adrián Galiana Rodríguez^3^, Shishir Baliyan^1^, César Venero Núñez^1^

^1^Department of Psychobiology, National University of Distance Education (UNED), 28040 Madrid, Spain

^2^Department of Psychology, Faculty of Health Sciences, Universidad Rey Juan Carlos, 28922 Madrid, Spain

^3^Department of Psychology, Faculty of Health Sciences and Education, Universidad a Distancia de Madrid (UDIMA), 28400 Madrid, Spain

**Introduction**: The *Val66Met* polymorphism of the BDNF gene has been associated with alterations in cortisol release and certain personality traits. However, evidence regarding its possible association with circadian cortisol rhythm and personality traits is scarce, and no studies to date have specifically focused on older adults. The hypothalamic-pituitary-adrenal (HPA) axis, and cortisol in particular, may serve as a psychobiological mechanism linking genetic predispositions to behavior. **Methodology**: We conducted an exploratory study to examine the relationship between the *Val66Met* polymorphism, daily cortisol release, and specific personality traits in women over 60 years old. The sample comprised 106 women (69 non carriers of the Met polymorphism and 37 Met carriers) and 30 men (17 non carriers of the Met polymorphism and 13 Met carriers). Salivary cortisol was measured at six time points across the day using and electronic monitoring device to ensure accurate recording of saliva collection, awakening time, and bedtime. Total cortisol output was calculated using the area under the curve (AUCtotal), and personality traits were assessed with the Ten-Item Personality Inventory (TIPI). Univariate general linear models were used, controlling for age, educational level, and depressive symptoms (Geriatric Depression Scale, GDS). **Results**: Results showed that women carrying the Met allele exhibited lower total daily cortisol release, as well as lower scores in agreeableness and conscientiousness. On the other hand, we did not find significant changes in men. Mediation analyses tested whether cortisol mediated the association between genotype and personality traits, but no significant indirect effects were observed. **Conclusions**: These findings suggest that the *Val66Met* polymorphism may be linked to both neuroendocrine function and personality in older women, although the mechanisms remain unclear. Further research is warranted to elucidate the role of cortisol regulation as a potential pathway linking genetic variation and behavioral phenotypes in aging.

**P30. Effectiveness of Cognitive-Behavioural Therapy for Coping With Stress in Patients With Systemic Sclerosis**

Eva Montero-López^1^, Ana Santos-Ruiz^2,3^, Raquel González-Pérez^4^, Norberto Ortego-Centeno^5,6^, María Isabel Peralta-Ramírez^7,8^

^1^Department of Developmental Psychology, University of Granada, 18071 Granada, Spain

^2^Department of Health Psychology, University of Alicante, 03690 Alicante, Spain

^3^Alicante Institute for Health and Biomedical Research (ISABIAL), 03010 Alicante, Spain

^4^Department of Pharmacology, University of Granada, 18071 Granada, Spain

^5^Department of Medicine, University of Granada, 18071 Granada, Spain

^6^Biosanitary Research Institute, IBS, 18012 Granada, Spain

^7^Mind, Brain, and Behavior Research Center (CIMCYC), University of Granada, 18071 Granada, Spain

^8^Department of Psychological Assessment and Treatment, University of Granada, 18071 Granada, Spain

**Introduction**: Systemic sclerosis (SSc) is a rare autoimmune disease that produces significant psychosocial impairment, making rehabilitation in this area essential. Its degenerative nature affects patients’ personal, occupational, and social lives, frequently leading to depression, anxiety, sleep disturbances, sexual dysfunction, and alterations in self-perception and body image due to the physical changes associated with the disease. Research on the role of psychological stress in autoimmune disorders has consistently demonstrated a close relationship between behavioral responses to stress and underlying neurophysiological and biochemical processes. In addition, psychological stress is recognized as an influential factor in both the onset and progression of systemic autoimmune diseases, with wide-ranging physical, emotional, and environmental consequences. Considering this evidence, the management of autoimmune conditions such as SSc should include psychological factors that may influence the course of the disease. One promising approach involves training patients in stress-management strategies through psychological interventions such as cognitive-behavioral therapy (CBT). CBT enables patients to address maladaptive thoughts and behaviors while developing effective coping mechanisms to reduce stress. **Methodology**: We conducted a study comparing pre- and post-intervention levels of hair cortisol, stress vulnerability, and bodily pain between two groups of patients with SSc: an intervention group receiving CBT and a control group without therapy. **Results**: Results indicated that patients in the CBT group showed significant reductions in hair cortisol levels, perceived stress vulnerability, and bodily pain compared to controls. **Conclusions**: These findings highlight the positive impact of CBT on both physiological and psychological indicators of stress in systemic sclerosis, reinforcing the importance of incorporating psychological interventions into comprehensive treatment programs for these patients.

**P31. Exploring the Use of Laughter for Early Neuro/Psychiatric Diagnosis and Evaluation**

I. Retuerta^1,2^, M. Terriza^3^, J. Navarro^1,2,3^, E. García^2,4^, P.C. Marijuán^1,2^, F. Panetsos^3^, N. Alfageme^3^, G. Kontaxakis^5^, R. San-Segundo^6^

^1^Bioinformation and Systems Biology Group, Aragon Institute of Health Science (IACS), 50009 Zaragoza, Spain

^2^Aragon Health Research Institute (IIS Aragón), 50009 Zaragoza, Spain

^3^Neurocomputing & Neuro-Robotics Research Group, Complutense University of Madrid, 28040 Madrid, Spain

^4^Department of Ophthalmology Research Group (GIMSO), Miguel Servet Hospital, 50009 Zaragoza, Spain

^5^Biomedical Image Technologies Group, Information Processing and Telecomunications Center, Politecnica de Madrid, 28040 Madrid, Spain

^6^Speech Tecnology Group, Information Processing and Telecomunications Center, 28040 Madrid, Spain

**Introduction**: In the medical field, laughter has been studied for its beneficial effects on health and as a therapeutic method to prevent and treat major medical diseases. However, very few works, if any, have explored the predictive potential of laughter and its potential use as a diagnostic tool. **Methodology**: One the one hand we registered laughs of depressed patients (n = 30) and healthy controls (n = 20), the processing was made in Matlab, general and discriminant analysis distinguished patients, controls, gender, and the association between laughter and HDRS test. On the other hand, we tested its efficacy in Parkinson’s disease (PD) evaluating different cepstral coefficients to identify laugh characteristics of healthy and ill subjects combined with machine learning classification models. **Results**: Depressed patients and healthy controls differed significantly on the type of laughter, with 88% efficacy. In the same way, the decision support system reached 83% accuracy rate with an AUC value of 0.86 for PD-healthy laughs classification. **Conclusions**: Laughter may be applied as a diagnostic tool in the onset and evolution of depression and, potentially, of neurodegenerative pathologies like PD. The sound structures of laughter reveal the underlying emotional and mood states in interpersonal relationships and also carries a significant neural and motor information.

**P32. Randomised Controlled Trial to Assess the Influence of a Fermented Dairy Product With Probiotics on Stress and Sleep Quality in Moderately Stressed Adults**

Isabel López-Chicheri^1^, Marina Iniesta-Sepúlveda^1^, Harry T. A. Moore^1^, Ana I. LópezNavas^1^, Antonio Ríos-Zambudio^2^, Bernadette Klotz^3^, Mary Luz Cano-Sepúlveda^3^, Mª Isabel Vasallo-Morillas^1^

^1^UCAM Universidad Católica de Murcia, 30107 Murcia, Spain

^2^Unidad de Trasplantes, Hospital Clínico Universitario Virgen de la Arrixaca, 30120 Murcia, Spain

^3^Alpina Productos Alimenticios S.A., Bogotá, Colombia

**Introduction**: In recent years, there has been a growing interest in the study of the gut-brain axis and the potential positive effects of probiotic intake on variables such as stress and sleep quality. The aim of the present study was to evaluate the efficacy of consuming a fermented dairy beverage containing Lactobacillus brevis DHe1_24_DWN on stress levels and sleep quality in moderately stressed healthy adults. **Methodology**: A total of 109 adults who reported being moderately stressed (according to the PSS-14 scale) and not adhering to the Mediterranean diet (as measured by the PREDIMED scale) participated in the study. Participants (64% women) were aged between 18 and 58 years (M = 28.28, SD = 11.47) and were randomly assigned to one of two study groups. Over a period of 8 to 9 weeks, they consumed either the probiotic product or a placebo beverage with no probiotic content. Sleep quality was assessed through nocturnal accelerometry over three nights following each visit, and via the Pittsburgh Sleep Quality Index (PSQI). Perceived stress levels were measured using a visual analogue scale (VAS). **Results**: For participants who consumed the functional product, a significant reduction in stress was observed between visit 1 and visit 2 (*p* < 0.05), as well as between visit 1 and visit 3 (*p* < 0.05). Differences in stress between treatment groups approached significance from visit 1 to visit 2 (*p* = 0.07). Regarding sleep quality, although improvements were observed in several parameters, none were statistically significant between groups. In the experimental group, sleep latency showed the greatest decrease, nearing significance between visit 1 and visit 2 (*p* = 0.08). **Conclusions**: The moderate stress levels and good baseline sleep quality of participants may have limited the detection of significant group differences. These findings suggest potential benefits that warrant confirmation in larger and longer-term studies.

**P33. The Positive Impact of Fetal Programming on Neurodevelopment After the COVID-19 Pandemic**

Lara Pérez Peregrín^1^, Miguel Ángel Baos-Gonlález^1,2^, Javier De Echarri-Lorente^1,2^, Raquel González-Pérez^3^, María Isabel Peralta-Ramírez^1,2^

^1^Mind, Brain and Behavior Research Center (CIMCYC), University of Granada, 18071 Granada, Spain

^2^Department of Personality, Assessment, and Psychological Treatments, Faculty of Psychology, University of Granada, 18071 Granada, Spain

^3^Department of Pharmacology, Centro de Investigación Biomédica en Red de Enfermedades Hepáticas y Digestivas (CIBERehd), School of Pharmacy, Instituto de Investigación Biosanitaria ibs. GRANADA, University of Granada, Granada, Spain

**Introduction**: The stress caused by the COVID-19 pandemic is known to have had consequences both on the psychopathology and stress levels of pregnant women and on the neurodevelopment of their offspring. Few studies have investigated the long-term consequences of the pandemic in this population. Therefore, the aim of this research was to determine whether there are differences in psychopathology and stress among pregnant women before and after the pandemic, as well as in the subsequent neurodevelopment of their offspring. **Methodology**: Two groups were included: a pre-pandemic group, with 163 mother–child dyads, and a post-pandemic group, with 109 mother–child dyads. **Results**: The results showed statistically significant differences between the two groups in the Perceived Stress Scale, hair cortisol concentration during pregnancy, and the Anxiety and Depression dimensions of the Symptom Checklist-90-R (SCL-90-R) in mothers. Regarding infant neurodevelopment, significant differences were found in the scaled score of the Fine Motor subscale, in the total and scaled scores of the Gross Motor subscale, in the scaled score of Motor Skills, and in the total score of the Expressive Communication subscale. **Conclusions**: Increased stress and psychopathology were observed among pregnant women after the pandemic, however, their offspring showed higher neurodevelopment scores post-pandemic.

**P34. Impact of Maternal COVID-19 Diagnosis and Anxiety on Early Child Neurodevelopment**

H. Bermejo-Pérez^1,2^, A. Mesquita^3^, R. Costa^4^ S. Dominguez-Salas^1,5^, S. Conejo-Cerón^6^, D. Gomez-Baya^7^, H.A. Andrade-Ruiz^2^, E. Motrico^1,5^

^1^Institute of Biomedicine of Seville (IBiS), 41013 Seville, Spain

^2^Foundation for Health Research Management in Seville (FISEVI), 41013 Seville, Spain

^3^ProChild CoLAB against Poverty and Social Exclusion, Campus de Couros, 4804-533 Guimarães, Portugal

^4^Faculty of Psychology, Education and Sport, University of Porto, 4200-135 Porto, Portugal

^5^Department of Developmental and Educational Psychology, University of Seville, 41004 Seville, Spain

^6^Institute of Biomedical Research of Malaga (IBIMA), 29010 Malaga, Spain

^7^Department of Social, Developmental and Educational Psychology, University of Huelva, 21007 Huelva, Spain

**Introduction**: Viral infection during pregnancy has been suggested to have an impact on offspring neurodevelopment, a process which may be mediated by the activation of immune response. Nevertheless, the association between maternal COVID-19 diagnosis and child neurodevelopment is still poorly understood. This cross-sectional observational study aimed to investigate the association between maternal COVID-19 diagnosis during pregnancy and/or postpartum, and child neurodevelopment between 18 and 35 months of age. **Methodology**: In this study, data from 419 Spanish mother–child dyads who gave birth during the COVID-19 pandemic were included. Variables assessed comprised sociodemographic characteristics, maternal COVID-19 diagnosis during pregnancy and/or postpartum, anxiety (measured using the Generalized Anxiety Disorder-7 scale, GAD-7) and child neurodevelopment (measured with the Caregiver Reported Early Development Instruments, CREDI). **Results**: The mean maternal age was 36.66 years (SD = 4.17), and the mean child age was 27.37 months (SD = 3.79). The prevalence of COVID-19 diagnosis was 10.98%. Generalized linear models (GLMs) with Gamma distribution were fitted to the data, including the five domains of CREDI (motor, cognitive, language, socio-emocional and overall) as outcome variables. For each domain, a model was adjusted for the following covariates: maternal COVID-19 diagnosis during pregnancy and/or postpartum, GAD-7 and age of the offspring. No significant associations were found between maternal COVID-19 diagnosis and child’s neurodevelopmental outcomes. By contrast, maternal anxiety was significantly associated with child’s cognitive and socio-emotional developmental domains. **Conclusions**: These findings suggest that maternal COVID-19 diagnosis may not directly affect early neurodevelopment after adjusting for relevant covariates. However, maternal anxiety may be associated with early neurodevelopmental outcomes, underscoring the potential role of modifiable risk factors, such as maternal mental health, in shaping early neurodevelopment.

## 4. Divulgation

**D1. Microbiota And Depression: A Silent Connection?**

Rocío Rodríguez Valdés^1^, Arian Izadi^2,3^, María Isabel Peralta Ramírez^3,4^

^1^Mind, Brain and Behavior Research Center (CIMCYC), University of Granada. 18071 Granada, Spain

^2^Oral Surgery and Implantology, Faculty of Dentistry, University of Granada, 18011 Granada, Spain

^3^Polisalud Clinics – Cristo de la Salud Polyclinic, 18220 Granada, Spain

^4^Department of Personality, Assessment and Psychological Treatment, Faculty of Psychology, University of Granada, 18071 Granada, Spain

The audiovisual content will address depression from an innovative perspective, integrating recent advances on the role of the gut microbiota in regulating neurogenesis and inflammatory processes. This resource aims to rigorously and comprehensibly explain how the interactions between the central nervous system, the immune system, and the intestinal microbial ecosystem shape new pathways to understand the pathophysiology of this disorder. First, depression will be introduced as one of the leading causes of disability worldwide, affecting millions of people and projected to become the primary global cause of disease burden by 2030. Its multifactorial nature will be highlighted, involving genetic, environmental, and biological factors. Next, the neurogenic hypothesis will be explained, which argues that a decrease in the formation of new neurons in the hippocampus is linked to depressive symptomatology and to the effectiveness of antidepressant treatments. Subsequently, the immune hypothesis will be presented, framing depression as an inflammatory disorder. Evidence will be shown on how increased levels of proinflammatory cytokines such as IL-1β, IL-6, and TNF-α correlate with core depressive symptoms, while also interfering with adult neurogenesis. In this context, microglia, the resident immune cells of the brain, play a central role given their dual function in modulating inflammatory responses and participating in neuroplasticity processes. The gut–brain axis (GB axis) will be the core of the audiovisual resource, showing how gut microbiota regulates brain function through immune, endocrine, and neural pathways. Evidence on intestinal dysbiosis and its impact on systemic inflammation and hippocampal neurogenesis will be reviewed, highlighting studies linking altered microbiota to greater vulnerability to depression. Finally, focus will be given to microbiota-mediated modulation of microglia, a key mechanism connecting peripheral inflammatory states with structural and functional brain changes.

